# Hand Washing and Related Cognitions Following a Brief Behavior Change Intervention During the COVID-19 Pandemic: a Pre-Post Analysis

**DOI:** 10.1007/s12529-021-10042-w

**Published:** 2021-11-29

**Authors:** Jan Keller, Dominika Kwasnicka, Lea O. Wilhelm, Noemi Lorbeer, Theresa Pauly, Antonia Domke, Nina Knoll, Lena Fleig

**Affiliations:** 1 Department of Education and Psychology, Freie Universität Berlin, Berlin, Germany; 2 Faculty of Psychology, SWPS University of Social Sciences and Humanities, Wroclaw, Poland; 3 NHMRC CRE in Digital Technology to Transform Chronic Disease Outcomes, Melbourne School of Population and Global Health, University of Melbourne, Melbourne, Australia; 4 Medical School Berlin, Berlin, Germany; 5 Department of Psychology, University of Zurich, Zurich, Switzerland

**Keywords:** Hand washing, Intention, Self-efficacy, Self-monitoring, Behavior change intervention, COVID-19

## Abstract

**Background:**

Effective hand washing (for at least 20 s, with water and soap) is one of the health behaviors protecting against infection transmissions. Behavior change interventions supporting the initiation and maintenance of hand washing are crucial to prevent infection transmissions. Based on the Health Action Process Approach, the aim of this research was to conduct a pre-post analysis of hand washing and related cognitions (i.e., intention, self-efficacy, self-monitoring), measured up to 100 days following an intervention.

**Methods:**

A convenience sample of *N* = 123 participants (age: *M* = 23.96 years; *SD* = 5.82; 80% women) received a brief intervention (key behavior change techniques: information about health consequences of hand washing; action planning) and responded to daily diaries and questionnaires up to a 100-day follow-up. Two-level models were used to analyze data of *n* = 89 participants who provided longitudinal data.

**Results:**

Hand washing and self-monitoring increased, whereas intention and self-efficacy decreased over time. Only self-monitoring was a consistent positive correlate of hand washing on a between-person level.

**Conclusions:**

Hand washing and self-monitoring considerably increased over several weeks following the intervention. Future research testing the intervention against a control condition is needed to rule out that changes in behavior and cognitions might have been prompted by completing the daily diaries.

**Trial Registration:**

German Clinical Trials Register; https://www.drks.de; registration number: DRKS00022067.

**Supplementary Information:**

The online version contains supplementary material available at 10.1007/s12529-021-10042-w.

## Introduction

### The COVID-19 Pandemic and Hand Washing

After the World Health Organization declared a COVID-19 pandemic on 11 March 2020 [1], COVID-19 incidence rates in Germany peaked in mid-March and were on a steady decline thereafter [2] with governmental restrictions applying from late March to May 2020. Throughout the summer, the German federal public health authority (Robert Koch Institute) reported a continuous and slow increase in incidence rates and relatively low mortality rates when compared to the situation in March [3]. From mid-October onwards, incidence rates in Germany showed a stronger increase [3] and mortality rates were rising [4].

During times of a global pandemic, protective health behaviors are of particular public interest, with health agencies and governments highlighting their importance in order to slow down the spread of SARS-CoV-2 infections [5]. Wearing facemasks, physical distancing, and effective hand hygiene are key to protect oneself and others from SARS-CoV-2 infections [5]. Recent evidence showed that SARS-CoV-2 survives on the human skin for several hours, longer than the influenza A virus, highlighting the importance of regular skin hygiene, such as effective hand washing [6, 7]. According to guidelines from the WHO, effective hand washing is defined as an act of cleaning hands for at least 20 s with water and soap (or equivalent materials), and in critical situations such as before preparing a meal, after coming home, or after arriving at work [8]. Data of the serial cross-sectional COVID-19 Snapshot Monitoring (COSMO) study show that in Germany in summer 2020, nearly 50% of respondents reported to either rarely or never wash their hands effectively or doing so, but not at every recommended occasion [9]. Thus, there is a particular need to promote effective hand washing (i.e., for at least 20 s, with water and soap) to remove SARS-CoV-2 virus from hand surfaces [7]. Effective hand washing is crucial; however, protective behaviors interrupting airborne transmission are also necessary to slow down the COVID-19 pandemic (e.g., wearing face masks). For the prevention of several gastrointestinal and respiratory infections, effective hand has important general value [6]. Since hand washing frequency is dependant on the occurrence of hand washing-relevant situations (e.g., upon entering home), variation in policy-level restrictions (e.g., working from home mandatory) during the COVID-19 pandemic can impact hand washing behaviors.

### Theoretical Underpinning of Behavior Change Interventions

Given the importance of effective hand washing, particularly in times of the COVID-19 pandemic, it is crucial that individuals who do not adhere to hand washing guidelines form intentions to do so and maintain hand washing over time [5, 10]. As a theoretical framework for behavior change, the Health Action Process Approach, HAPA [10], distinguishes between motivational processes that lead to a behavioral intention and volitional processes that support the translation of intentions into action [10]. Regarding motivational processes, explaining how to perform health behaviors effectively and how to practically embed the behavior in daily life could increase the likelihood that individuals form intentions, such as washing hands for at least 20 s with water and soap [11]. With respect to volitional processes, planning of health behavior (i.e., action planning) helps individuals to act in accordance with their intentions and bridges the so-called intention-behavior-gap [12]. As one form of action planning, individuals could plan daily life situations relevant to effective hand hygiene (e.g., after coming home) that serve as prompts to engage in hand washing, whenever the daily life situation occurs. Action planning has been shown to be effective in leading not only to an increase in the planned behavior [12], but also to increased self-efficacy levels [13, 14]. Planning facilitates reaching behavioral intentions by breaking down a distal goal into proximal plans, which in turn reduces barriers and increases the belief in one’s competence to enact the plan [13]. Moreover, action planning may lead to stronger self-monitoring, i.e., monitoring the progress towards the hand washing plan by, e.g., reminding yourself whether hands were washed after coming home [15, 16]. The HAPA model was a key theoretical underpinning of the tested intervention; however, we also acknowledge that tested theoretical constructs are also included in other theoretical models that emphasize self-efficacy (e.g., Social Cognitive Theory [17]) and self-monitoring (e.g., Self-monitoring Theory [18]).

### Fostering Hand Washing Through Theory-Based Behavior Change Interventions

Behavioral scientists emphasize that health promotion campaigns aiming to foster hand washing should avoid messages based on fear or disgust in relation to other people as they could undermine self-efficacy [19]. In contrast, resource-oriented health promotion programs that include educational materials and explain risk situations for disease transmission are a promising mean to foster people’s intention to wash hands [19]. Once motivated to wash hands, interventions could support individuals in translating their intentions into action by promoting hand washing–related cognitions (e.g., self-efficacy), using self-regulatory strategies such as action planning or setting prompts/cues [20].

Existing hand washing behavior change interventions usually examined hand washing up to a short-term follow-up (e.g., 1 month) and showed that the combined use of educational material and self-regulatory strategies is effective to foster hand washing [20] and self-efficacy [14, 20]. To examine whether behavior change interventions are associated with not only an initial increase in hand washing, but also with its maintenance, more research with longer-term follow-ups is needed. Moreover, examining associations over time (i.e., immediately following the intervention, but also at longer-term follow-ups) between hand washing–related cognitions and hand washing is needed to learn more about hand washing interventions [20].

Evidence from a large randomized controlled trial revealed that a hand washing behavior change intervention can also prevent infection transmission in the long-term [11, 21]. Little and colleagues [11] concluded that simple, online, and scalable interventions show great potential, particularly in times of pandemics.

### Aim and Hypotheses

**Fig. 1:**
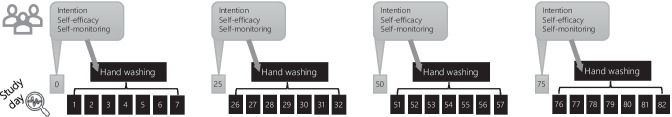
Four assessments of hand washing–related cognitions predicting next-week hand washing

In this study, a German convenience sample was recruited, a brief behavior change intervention to foster hand washing was conducted, and hand washing and related cognitions were examined up to a 100-day follow-up. To extend previous intervention studies focusing on short-term effects, e.g., [14, 20], both short-term and longer-term trajectories (i.e., up to a 100-day follow-up) are reported in the present study. The theory-based intervention included educational material and applied self-regulatory strategies, such as planning prompts/cues for effective hand washing (i.e., for at least 20 s, with water and soap) in highly relevant situations. The behavior change intervention also aimed to foster hand washing–related cognitions, including the intention to wash hands for at least 20 s as well as hand washing–related self-efficacy and self-monitoring. To examine correlates of hand washing at different follow-up time points, within- and between-person relationships of repeatedly assessed hand washing–related cognitions (i.e., at baseline and at 25-day, 50-day, and 75-day follow-ups) with next-week hand washing (i.e., from assessments of the next 7 days, respectively) were also investigated (Fig. 1). Three main study hypotheses were as follows:

- Hypothesis 1: Hand washing will increase over time following the intervention.
- Hypothesis 2: Hand washing intentions (H2a), self-efficacy (H2b), and self-monitoring (H2c) will increase following the intervention.
- Hypothesis 3: Next-week hand washing will show positive associations with hand washing–related intentions (Hypothesis 3a), self-efficacy (Hypothesis 3b), and self-monitoring (Hypothesis 3c) over time. To examine effects across the study period (between-person level) as well as effects on the assessment level (within-person level), between- and within-person predictions of intention, self-efficacy, and self-monitoring should be analyzed [22].

## Method

### Participants

In July 2020, the study was advertised to university students and staff of the Freie Universität Berlin and Medical school Berlin through email lists and online postings. Participants were offered an online shopping voucher of 5 EUR for completing the 100-day study period and, if applicable, course credits. Eligible participants were at least 18 years old and had sufficient comprehension of the German language and ability to understand and complete the study materials. A convenience sample of *N* = 123 participants from Germany provided consent and completed the baseline questionnaire. The participants (98 women; 80%) had a mean age of 23.96 years (*SD* = 5.82; range: 18–48), and 13 of them (11%) reported to live together with their child/children. Whereas 21 participants (17%) lived alone, the remaining participants (*n* = 102; 83%) lived in a household consisting of 2–10 persons, with the majority (*n* = 93) living in a household of 2–4 persons. At baseline (July 2020), *n* = 10 participants (8%) reported flu-like symptoms with one participant being in COVID-related quarantine.

### Procedure

This study was preregistered in the German Clinical Trials Registry (DRKS-ID: DRKS00022067). The present article reports secondary analyses of this study. The primary aim of the overall study was to examine persons’ hand washing–related habit formation (primary outcome: automaticity of hand washing) across a daily diary period up to a 86-day follow-up as well as to investigate habitual maintenance up to a 100-day follow-up.

Data collection was conducted between July 12, 2020, and November 02, 2020. In this period, daily COVID-19 incidence rates were relatively low in Germany at the start of the study (e.g., 248 new cases on July 12, 2020), followed by increases over time up to very high incidence rates at the end of the study (e.g., 12,097 new cases on November 02, 2020). More details on the pandemic situation in Germany during times of data collection of the present study are included in Electronic Supplementary Material 1.

After responding to the baseline questionnaire (Day = “D”; D0), participants received a brief intervention aiming to promote participants’ hand washing by asking them to choose up to two situations of their daily life in which they want to form a new hand washing habit. The intervention was framed as the *100-day hand washing challenge*. Over the subsequent 86 days, participants were asked to respond to brief end-of-day questionnaires (daily diary period; D1–D86), that included items on daily hand washing and characteristics of participants’ planned situations. Additionally, participants completed longer questionnaires at 25 (D25), 50 (D50), 75 (D75), and 100 (D100) days following the baseline and intervention session, which included measures of hand washing–related cognitions (e.g., intention, self-efficacy, and self-monitoring). The institutional review board of the Medical School Berlin (MSB-2020/36) granted ethics approval for this study.

The questionnaire completion adherence was relatively high, *n* = 88, *n* = 77, *n* = 69, and *n* = 81 participants provided data at D25, D50, D75, and D100, respectively. Overall, a total of *n* = 89 (out of *n* = 123: 72%) participants provided longitudinal data throughout the daily diary and questionnaire period; this sub-group is therefore used as the sample of analysis for the present research questions.

### Intervention

The brief online intervention was based on a previous theory-based intervention [23]. The intervention material is provided in the Electronic Supplementary Material 2. Behavior change techniques (BCTs) applied in the present intervention are listed below [24]. Using educational material in the first part of the intervention, participants received general information about effective hand washing (for at least 20 s, with water and soap), its pros and cons (BCT 9.2), and information about health consequences of hand washing (BCT 5.1), which was linked with the COVID-19 pandemic. They also received instructions on how to perform the behavior (BCT 4.1), illustrated by photographs of effective hand washing. Participants were asked to write down what can make hand washing feel good or pleasant (e.g., using soap which smells nicely; BCT 10.7: self-incentive).

The second part of the intervention comprised the BCTs prompts/cues (BCT 7.1), action planning (BCT 1.4), self-monitoring of behavior (BCT 2.3), and habit formation (BCT 8.3). Participants created their personalized hand washing plan by writing down up to two situations of their daily life (i.e., prompts/cues) in which they would like to form a new hand washing habit. The cues could refer to anything that can be experienced in daily life but occurs (a) several times a week and (b) with a certain degree of regularity. To increase adherence to the protocol and to support effective hand hygiene, four cue examples relevant to SARS-CoV-2 transmission prevention were provided (e.g., “before preparing a meal”). To foster self-monitoring, participants were instructed to write down or photograph the cues specified in the online tool. Finally, they were asked to wash hands, whenever their planned cues will occur throughout the following days. This was framed as their “100-day hand washing challenge.”

### Measures

#### Effective Hand Washing

Present measures for effective hand washing (henceforth described as hand washing) refer to the frequency of hand washing (times per day), which is done for at least 20 s with water and soap [8]. To examine the present hypotheses, two hand washing measures were used. Using daily end-of-day reports, (1) *daily hand washing* was assessed throughout D0 to D86 using the item “How often did you wash your hands for 20 s with water and soap today?” When participants missed an end-of-day response, they could retrospectively report it on the next day. For analyses, testing associations between hand washing–related cognitions and subsequent hand washing, (2) a *next-week hand washing* variable was computed reflecting daily mean levels across the 7 days following the questionnaire assessments (i.e., D1–D7, D26–D32, D51–57, and D76–D82).

#### Hand Washing–Related Cognitions

Intention, self-efficacy, and self-monitoring were assessed at D0, D25, D50, D75, and D100 using the response format 1 = “does not apply at all” to 6 = “applies exactly.” Items were adapted from an earlier study examining hand washing–related cognitions [25]. *Intention* was assessed with the item “For tomorrow I intend the following: Each time I wash my hands, I will wash my hands for at least 20 s.” *Self-efficacy* was measured using the item “I am confident I can wash my hands even in difficult situations (e.g., when I am in a hurry),” note that the 20-s specification was not included within this measure. *Self-monitoring* was assessed with the item “In the past 7 days, I regularly monitored if I washed my hands for at least 20 s.”

#### Covariates

Baseline covariates were participants’ age, sex, experiencing flu-like symptoms, number of persons in the household, and working in home office. As an additional covariate, *risk perception* at D0, D25, D50, D75, and D100 was included, assessed with the item “How likely is it for you to become infected with coronavirus SARS-CoV-2 without washing your hands regularly?” on a scale of 1 = “very unlikely” to 5 = “very likely.” Preliminary analyses showed that negative outcome expectancies (assessed at baseline: “If I wash my hands regularly, then it would take too much of my time”) was an attrition mechanism and therefore added to the list of covariates.

### Statistical Power

Sample size calculation for primary analyses (outcome: automaticity over time) was performed using G*Power v3.1.9.7. Based on prior studies [23, 26], a large effect of *f* = 0.4 and an autocorrelation of 0.85 were assumed. A power of 0.80 and 87 repeated measurements resulted in a required sample size of *n* = 89.

For secondary analyses as carried out for the present article, we conducted a post hoc power analysis using the SIMR package in R [27]. We assumed effect sizes typical for behavioral research, 0.2 for fixed effects of predictors of interest [28] and random effects to generate 1000 simulations (Monte Carlo method). For the analysis of Hypothesis 1, which was based on 86 days nested within 89 participants, there was 97% power to detect a time-trend in hand washing behavior. Regarding analyses of Hypothesis 2, which were based on five observations nested within 89 participants, there was 82 to 83% power to detect temporal changes in hand washing–related cognitions. For Hypothesis 3 analyses, which were based on four observations nested within 89 participants, there was 67 to 86% power to detect associations between hand washing–related cognitions and hand washing behavior at the within-person level and 65 to 69% power to detect associations at the between-person level.

### Analyses

All reported data analyses were conducted with the R software, Version 4.0.3, and IBM SPSS 28. Data analysis scripts can be obtained from the authors upon request.

#### Preliminary Analyses

To examine whether the sample of analysis (*n* = 89) differed from those who only provided baseline data (*n* = 34), differences between baseline variables and a dichotomous attrition variable (0 = not retained for analysis, 1 = retained) were examined using *χ*^2^- and *t*-tests, followed by logistic regressions. To explore the time trend of COVID-19 incidence rates throughout the study period, COVID-19 incidence rates in Germany were extracted from a public database [2], matched with dates of data collection, and study assessment-incidence rate correlations were computed.

#### Change Over Time in Study Variables

To test for changes in *daily hand washing* throughout D1 to D86, two-level models of a person-period dataset with 86 days nested in participants were run in SPSS allowing for first-order autoregressive structure with homogenous variance [29]. The linear study day trend, centered at D1 = 0, was included as a random effect predictor of daily hand washing (i.e., modeling that hand washing varied between persons [30]). A quadratic time trend was explored in additional analyses but showed no significant effect on daily hand washing and was therefore excluded from further analyses. Sensitivity analyses examined whether linear time predictions held when controlling for a weekday variable (0 = weekend day; 1 = weekday) and daily COVID-19 incidence rates.

Change over time in *intention*, *self-efficacy*, and *self-monitoring* across D0, D25, D50, D75, and D100 were examined in SPSS using repeated-measures ANOVAs and *t*-tests between subsequent measures.

#### Multivariate Associations Between Hand Washing–Related Cognitions and Next-Week Hand Washing

In order to create between- and within-person components, repeated assessments of hand washing–related cognitions were grand-mean and then person-mean centered. Within-person correlations between intention, self-efficacy, self-monitoring, and next-week hand washing were examined using the *rmcorr* package in R [31]. To test Hypothesis 3, two-level models with four assessment periods nested in participants were estimated in SPSS, allowing for first-order autoregressive structure with homogenous variance [29]. In a model including all hand washing–related cognitions and covariates simultaneously, intention, self-efficacy, and self-monitoring were modeled at the between-person level (e.g., comparing persons with higher average versus lower average hand washing intentions) and the within-person level (e.g., assessments when intentions to wash hands were higher-than-usual vs. lower-than-usual). A linear time predictor (assessment following D0; 0 = D0, 1 = D25, 2 = D50, 3 = D75) was added. Again, a quadratic time prediction was tested, but due to non-significant links with the hand washing outcome, the quadratic time prediction was removed from further analyses. Stepwise, possible random effects were tested and retained as long as models converged [30]. Additionally to the random intercept, the random effect of within-person self-efficacy was modeled. No other random effects could be modeled due to issues of model non-convergence.

Missing data were treated using restricted maximum likelihood procedure (REML).

## Results

### Preliminary Analyses

After being instructed in the intervention to choose situations for a new hand washing habit, most often selected situations were (a) after coming home (22 participants; out of 89: 25%), (b) after (longer) smartphone use (20 participants; 22%), (c) before (preparing) a meal (19 participants; 21%), and (d) before snacking (10 participants; 11%). The remainder includes rarely selected situations such as “before using cosmetics” and “after sneezing.”

Compared with participants who only provided baseline data (*n* = 34), participants retained for analyses (*n* = 89) did not differ in study variables assessed at baseline, except for negative outcome expectancies which were higher in retained participants (not retained participants: *M* = 1.88, *SD* = 1.01; retained participants: *M* = 2.27, *SD* = 0.89; *t*(121) =  − 2.08, *p* = 0.039). For *n* = 89 participants (sample of analysis), the average response rate to daily diary prompts was 68.13 (out of 86 days: 79%; *SD* = 18.15; range: 13 to 86). Regarding the response rate for questionnaires following baseline (D25, D50, D75, and D100), 72% (*n* = 64 participants) of the sample of analysis responded to all questionnaires, whereas the remainder missed one (11%), two (9%), or three (8%) assessments. Missing analyses indicated significant Little’s MCAR tests for hypothesis 1, 2, and 3 analyses; that is, the missing completely at random assumption could not be confirmed for present analyses.

During the present data collection, COVID-19 incidence rates showed a continuous increase over time (Electronic Supplementary Material 1). For study dates corresponding to hand washing from D1 to D7, D26 to D32, D51 to D57, and D76 to D82, a high positive correlation (*r* = 0.88, *p* < 0.001) between 7-day incidence rates in Germany and a linear time trend was observed. Due to issues of multicollinearity, statistical models testing associations between hand washing–related cognitions and next-week hand washing only controlled for the linear time trend, but not the 7-day incidence rate.

### Change Over Time in Study Variables

#### Hand Washing

**Fig. 2:**
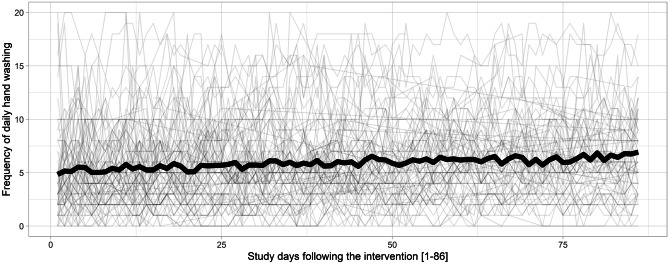
Spaghetti plot of hand washing frequency of *n* = 89 participants across the daily diary period (D0-D86). Note. Curve in bold reflects mean levels of respective study days

Across the daily diary period (D1 to D86), the intraclass correlation (*ICC*) of *daily hand washing* was 0.72, 95% CI (0.66; 0.78), indicating that most differences in daily hand washing were due to between-person differences and some were due to within-person fluctuations. Figure 2 displays all available data of participants’ daily hand washing and shows how hand washing can differ between persons, but also how it fluctuates over time within persons. Confirming Hypothesis 1, hand washing significantly increased throughout the study period (*b* = 0.02, *SE* = 0.01, 95% CI [0.01; 0.03], *p* < 0.001; Fig. 2). Specifically, participants on average washed their hands about 1.9 times more per day on D86, as compared with D0 (D0: 5.0 times; D86: 6.9 times), which represents a large effect size (*λ* = 0.84, *F* (1,63) = 11.85, *p* = 0.001; *η*^*2*^ = 0.16). The pattern of results holds when additionally controlling for weekend days and daily incidence rate in Germany. Hand washing was less likely at weekend days when compared to weekdays (*b* =  − 0.26, *SE* = 0.08, 95% CI [− 0.42; − 0.10], *p* = 0.001).

#### Hand Washing–Related Cognitions

In addition to time trajectories of the key hand washing–related cognitions examined in this article (i.e., intentions, self-efficacy, and self-monitoring), time trends of further HAPA-based cognitions (i.e., positive and negative outcome expectancies, risk perception, planning) are displayed in the Electronic Supplementary Material 3. Only positive outcome expectancies changed over time (decrease from D0 to D25), whereas the remaining motivational HAPA-based cognitions and planning remained stable over time. Regarding the hypothesized cognitions and contrary to Hypothesis 2a, participants’ intentions to wash hands for 20 s showed a continuous decrease throughout the study period, as indicated by a negative linear time trend (Table 1). A particularly pronounced decrease in intentions was observed between D50 and D75 (*t*(65) = 2.17, *p* = 0.034). Also contrary to the present assumptions (Hypothesis 2b), self-efficacy showed an initial decrease between D0 and D25 (*t*(87) = 3.13, *p* = 0.002), followed by a maintenance after D25. In line with Hypothesis 2c, self-monitoring increased between D0 and D25 (*t*(87) =  − 4.19, *p* < 0.001) and elevated self-monitoring levels were maintained after D25.

**Table 1:** Descriptive statistics of hand washing–related cognitions and hand washing

	**D0**	**D25**	**D50**	**D75**	**D100**	**Overall change over time**	
Hand washing–related cognitions	*M* (*SD*)	*M* (*SD*)	*M* (*SD*)	*M* (*SD*)	*M* (*SD*)	Wilks *λ*	Effect size
Intention (1–6)	4.64 (1.09)	4.39 (1.12)	4.32^a^ (1.17)	4.03^a^ (1.20)	4.05 (1.21)	Decrease: *λ* = .77, *F* (4,59) = 4.41, *p* = .003	*η*^2^ = .23
Self-efficacy (1–6)	4.45^b^ (0.87)	4.14^b^ (1.02)	4.04 (1.01)	4.12 (1.19)	4.27 (1.19)	Decrease: *λ* = .84, *F* (4,60) = 2.77, *p* = .035	*η*^2^ = .16
Self-monitoring (1–6)	3.65^c^ (1.32)	4.30^c^ (1.07)	4.11 (1.15)	4.21 (1.15)	4.26 (1.22)	Increase: *λ* = .82, *F* (4,60) = 3.37, *p* = .015	*η*^2^ = .18

68 ≤ *n* ≤ 89 participants due to missing values. “D” refers to day of assessment following baseline. Significant between-assessment differences, ^a^: *t*(65) = 2.17, *p* = .034; ^b^: *t*(87) = 3.13, *p* = .002; ^c^: *t*(87) =  − 4.19, *p* < .001

*M* mean, *SD* standard deviation

### Bivariate Associations Between Cognitions and Hand Washing

For assessments displayed in Fig. 1, bivariate correlations between intention, self-efficacy, self-monitoring, and next-week hand washing can be found in Table 2. Regarding within-person correlations, at assessments when participants reported higher-than-usual intentions to wash hands, they were more likely to report higher-than-usual self-efficacy (*r* = 0.21, *p* = 0.001). Moreover, at assessments when participants reported higher-than-usual self-monitoring, they were more likely to report more-than-usual next-week hand washing (*r* = 0.17, *p* = 0.013). At the between-person level (i.e., across the study), all hand washing–related cognitions were positively inter-related. Average next-week hand washing was higher when participants generally reported higher intentions to wash hands (*r* = 0.21, *p* = 0.045) and self-monitoring (*r* = 0.38, *p* < 0.001).

**Table 2:** Bivariate within- and between-person correlations between hand washing–related cognitions and hand washing

	Within-person			Between-person		
Variable	**2**	**3**	**4**	**2**	**3**	**4**
1. Intention	.21**	.07	− .05	.26*	.39***	.21*
2. Self-efficacy		.02	.03		.41***	.19
3. Self-monitoring			.17*			.38***
4. Next-week hand washing						

*n* = 89 participants. *t* = 4 occasions. Significant correlations in bold

*p < .05; **p < .01; ***p < .001

### Multivariate Cognition-Hand Washing Associations

To test Hypothesis 3, we entered hand washing–related cognitions measured at D0, D25, D50, and D75 as predictors in a model that estimated next-week hand washing. Table 3 presents results of two-level models (i.e., assessments nested in persons) predicting next-week hand washing and including the set of covariates. Additional results from a corresponding model with most often selected hand washing situations from the intervention as additional covariates are listed as Electronic Supplementary Material 3.

**Table 3:** Fixed effects estimates for two-level models predicting hand washing, with covariates

**Outcome: next-week hand washing**			
**Fixed effects**	***B* (*SE*)**	***p***	**95% CI**
Intercept (at study start, D0)	4.96 (0.50)	< .001	3.97; 5.94
Time (assessment following D0)	**0.40 (0.11)**	** < .001**	0.19; 0.61
Within-person intention	−0.05 (0.10)	.635	−0.26; 0.16
Between-person intention	0.51 (0.41)	.221	−0.31; 1.33
Within-person self-efficacy	0.20 (0.16)	.212	−0.12; 0.51
Between-person self-efficacy	−0.06 (0.44)	.892	−0.94; 0.82
Within-person self-monitoring	0.14 (0.09)	.135	−0.04; 0.33
Between-person self-monitoring	**1.02 (0.41)**	**.016**	0.20; 1.84
Age	0.10 (0.06)	.098	−0.02; 0.21
Sex (0 = female; 1 = male)	−0.64 (0.84)	.452	−2.32; 1.04
Flu-like symptoms	−1.76 (1.27)	.170	−4.29; 0.77
Number of persons in household	−0.31 (0.24)	.196	−0.78; 0.16
Working in home office	0.91 (0.67)	.178	−0.43; 2.26
Negative outcome expectancies at D0	−0.13 (0.38)	.734	−0.88; 0.63
Within-person risk perception	−0.15 (0.21)	.469	−0.55; 0.26
Between-level risk perception	0.66 (0.63)	.297	−0.59; 1.92
**Random effects**	**Variance (SE)**	***p***	
Intercept (at study start, D0)	2.33 (8.11)	.773	
Within-person self-efficacy	0.48 (0.30)	.115	

Analyses refer to *n* = 88 participants with 310 observations due to a missing value of one participant on the sex variable. Significant predictions in bold. A maximized random effect structure was modeled

*B *unstandardized estimate*, SE* standard error, *CI* confidence interval

Not in line with within-person assumptions of Hypothesis 3 (i.e., at assessments when cognition predictors were higher than usual), intention, self-efficacy, and self-monitoring were unrelated to next-week hand washing (Table 3). Not in accordance with between-level assumptions of Hypothesis 3, between-person intention (Hypothesis 3a) and self-efficacy (Hypothesis 3b) were not uniquely related to hand washing. In line with the hypothesized between-level relationship (Hypothesis 3c), individuals who generally reported higher self-monitoring reported higher next-week hand washing (*b* = 1.02, *SE* = 0.41, 95% CI [0.20; 1.84], *p* = 0.016). The coefficient of the between-person self-monitoring prediction (Table 3) reflects that persons with heightened self-monitoring throughout the study (one unit above the sample average) showed higher average next-week hand washing (higher by 1.02 times per day; Table 3). Moreover, covariates were not associated with hand washing, except the “after (longer) smartphone use” variable (*b* = 1.70, *SE* = 0.79, 95% CI [0.12; 3.28], *p* = 0.035; Electronic Supplementary Material 3). That is, participants who planned to wash hands after (longer) smartphone use in the intervention were more likely to wash hands more frequently.

## Discussion

The overall aim of present pre-post analyses was to investigate trajectories of hand washing and related cognitions following a brief online behavior change intervention (key BCTs: information about health consequences of hand washing; action planning) aiming to increase the frequency of effective hand washing (for at least 20 s, with water and soap). In line with Hypothesis 1, positive linear changes in hand washing following the intervention were observed. At the start of the study, participants reported an average frequency of hand washing of about five times per day. Following the intervention, they continuously increased their hand washing levels to around seven times per day at the 86-day follow-up. Our findings are in line with evidence from intervention studies showing that the use of theory-based BCTs, such as setting suitable prompts or self-monitoring of hand washing, can be a promising and time-effective mean to promote general hand washing [20]. As this brief and simple online intervention was followed by an increase in hand washing over several weeks, a future study could scale the present intervention and test it in a representative sample, and use a waiting-list control group to evaluate the effectiveness of the intervention. Also, infection transmission outcomes could be examined [11].

We also hypothesized (Hypothesis 2) that hand washing–related intentions (H2a), self-efficacy (H2b), and self-monitoring (H2c) would increase over time following the intervention. Contrary to H2a, intentions declined following the intervention, particularly in the second half of the study period (i.e., after D50). At times when participants already performed increased daily hand washing (i.e., on average around six times per day at D50), some participants might have lowered their hand washing intentions as hand washing frequency might have already been optimized for their daily life. Regarding self-efficacy levels, a decrease after the intervention was found, followed by a maintenance (contrary to H2b). Effective hand washing (i.e., for at least 20 s, with water and soap) across several daily life situations may be difficult to adhere to over time. After initially attempting to wash hands more frequently, participants might have experienced barriers impeding hand washing and might have decreased their self-efficacy beliefs within the first weeks following the intervention [32]. Confirming Hypothesis 2c, increases in self-monitoring were found, which is in line with previous studies suggesting that interventions targeting self-regulatory strategies are connected with improved self-monitoring [33]. Increases in self-monitoring early on following an action planning intervention are likely to be related to observing the target behavior in order to start one’s plan pursuit with full adherence [34].

Furthermore, positive associations of hand washing–related intentions, self-efficacy, and self-monitoring with hand washing over time were hypothesized (Hypothesis 3). To account for lagged effects of predictor-outcome relationships [35], we tested associations between hand washing–related cognitions measured at four assessments (D0, D25, D50, and D75) and aggregated daily reports of next-week hand washing (D1–D7, D26–D32, D51–D57, and D76–D82). Correlation analyses point towards low positive significant associations of hand washing with between-person intentions (*r* = 0.21, *p* < 0.05), whereas within- and between-person self-efficacy and within-person intentions did not show significant associations. When tested competingly with self-monitoring, within-person and between-person intentions and self-efficacy were not linked to next-week hand washing (not in line with Hypotheses 3a and 3b). As the present intervention particularly targeted participants’ plan pursuit and its day-by-day monitoring, intentions and self-efficacy beliefs might have played a minor role for hand washing. Only between-person self-monitoring was a consistent and positive hand washing correlate, indicating that participants with higher self-monitoring throughout the study period were more likely to report higher next-week hand washing. This finding is in line with previous research [25, 36] highlighting the importance to promote individuals’ continuous self-monitoring by interventions and its role as the most proximal predictor of engagement in health behaviors [16]. Not in line with our assumptions and when adjusting for covariates, a non-significant association between within-person self-monitoring and next-week hand washing was observed. That is, on time points when self-monitoring was higher-than-usual, no significant elevated levels of subsequent hand washing were found. Prediction strength of within-level associations is not always superior to between-level associations of the same constructs [37]. Recent methodological articles focusing on this phenomenon concluded that the temporal resolution matters [35, 37]. As within-person effects are referring to lagged analyses in the present study, issues of shared method variance of same-time analyses are circumvented [35]. This is a strength of the present study and a clear temporal order between variables (e.g., today’s self-monitoring is examined as a correlate of hand washing of the next 7 days) is investigated. Present non-significant within-person relationships could be explained by the time lag spanning one to seven days. A future daily diary study could measure cognitions in morning diaries and daily hand washing in evening diaries, which enables to examine lagged effects within the same day.

In present analyses, a number of variables were controlled for, that could potentially alter the hand washing outcome by providing more or less opportunities for hand washing. Among the situations that were planned in the intervention, only the situation “after (longer) smartphone use” was significantly related to hand washing frequency. Possibly a situation occurring multiple times a day provides more opportunities to act and thus contributed to more hand washing per day.

The present study had several strengths and limitations that need to be acknowledged. In terms of strengths, we used a study design that allowed examining within-person changes in hand washing following an intervention. To inform future hand washing interventions, hand washing–related cognitions addressed by the intervention were investigated as correlates of hand washing [38]. As hand washing varies from person to person, understanding reasons for individual differences will allow designing individually tailored precision interventions [24]. A relatively long study duration during the COVID-19 pandemic allowed us to test how hand washing changed throughout approximately 3 months of the pandemic. Another strength of the present study was the investigation of lagged effects between hand washing–related cognitions and a daily-assessed next-week outcome [35].

The present study also had several limitations. The sample was not representative of the general population and included relatively young participants, most of them highly educated. The analyzed sample of *n* = 89 participants provided sufficient statistical power to detect change of study variables over time; however, a larger sample size would have been desirable to detect within- and between-person associations between hand washing–related cognitions and hand washing. We only explored behavior change of hand washing during a certain period of time during the COVID-19 pandemic. Studies with data collection at different phases of the COVID-19 pandemic could bring different results. Regarding the hand washing measure, we chose to use a parsimonious 1-item measure to assess hand washing frequency to reduce daily participant burden. An additional behavioral measure capturing adherence to WHO-recommended hand washing situations would be desirable. Although present single-item measurements might have reduced participant burden, such a procedure comes along with limitations on validity and reliability (e.g., no parameters on internal consistency). Other than for intention and self-monitoring, the self-efficacy measure did not include an “at least 20 s” hand washing statement; thus, relationships between self-efficacy and hand washing should be interpreted with caution. Lastly, the study included pre-post analyses of an intervention. Future studies could explore if a no-intervention control condition also changes over time due to simply responding to the study questionnaires [39]. To make causal inferences on the present intervention and its mechanisms, a future randomized controlled trial could compare the present intervention with a control condition (e.g., waiting-list or education only) and investigate hand washing–related cognitions (e.g., self-monitoring) as mediators between the intervention and subsequent hand washing over time.

## Conclusions

Hand washing and hand washing–related self-monitoring increased over several weeks following this brief online intervention. The intervention was cost-effective as it was delivered online and self-guided by the intervention user. It was theory-based (HAPA model [10]) and included active ingredients of effective interventions. A future randomized controlled trial could compare the intervention applied in the present study with a control condition to assess between-group effects. This online intervention could be easily scaled to large and representative samples, and infection transmission outcomes could be further examined. In the time of current COVID-19 pandemic and potentially facing the threat of future pandemics, psychologists and behavioral scientists valuably contribute to the development of effective behavioral interventions such as this one. Providing interventions that can be easily implemented gives the opportunity to apply behavior change knowledge from health psychology to change hand washing at scale and to slow down virus spread.

## Supplementary Information

Below is the link to the electronic supplementary material.

Supplementary file1 (DOCX 47 KB)

Supplementary file2 (PDF 5274 KB)

Supplementary file3 (DOCX 72 KB)

## Data Availability

The datasets generated during this study are not publicly available as we do not have permission from study participants. However, group-level information about the data are available from the corresponding author on reasonable request. An English version of the intervention materials (originally in German) is included in the Electronic Supplemental Material 2 associated with this article. Data analysis scripts can be obtained from the authors upon request.
