# Supplementary material for: Hand Washing and Related Cognitions Following a Brief Behavior Change Intervention During the COVID-19 Pandemic: a Pre-Post Analysis
Source: Int J Behav Med. 2021 Nov 29;29(5):575–86. doi: 10.1007/s12529-021-10042-w (PMC8628490; doi:10.1007/s12529-021-10042-w)
Supplement: Supplementary file 1 — Supplementary file1 (DOCX 47 KB) [file 12529_2021_10042_MOESM1_ESM.docx]

**Electronic Supplementary Material 1:** COVID-19 situation in Germany in 2020 and data collection of the present study (marked in purple).


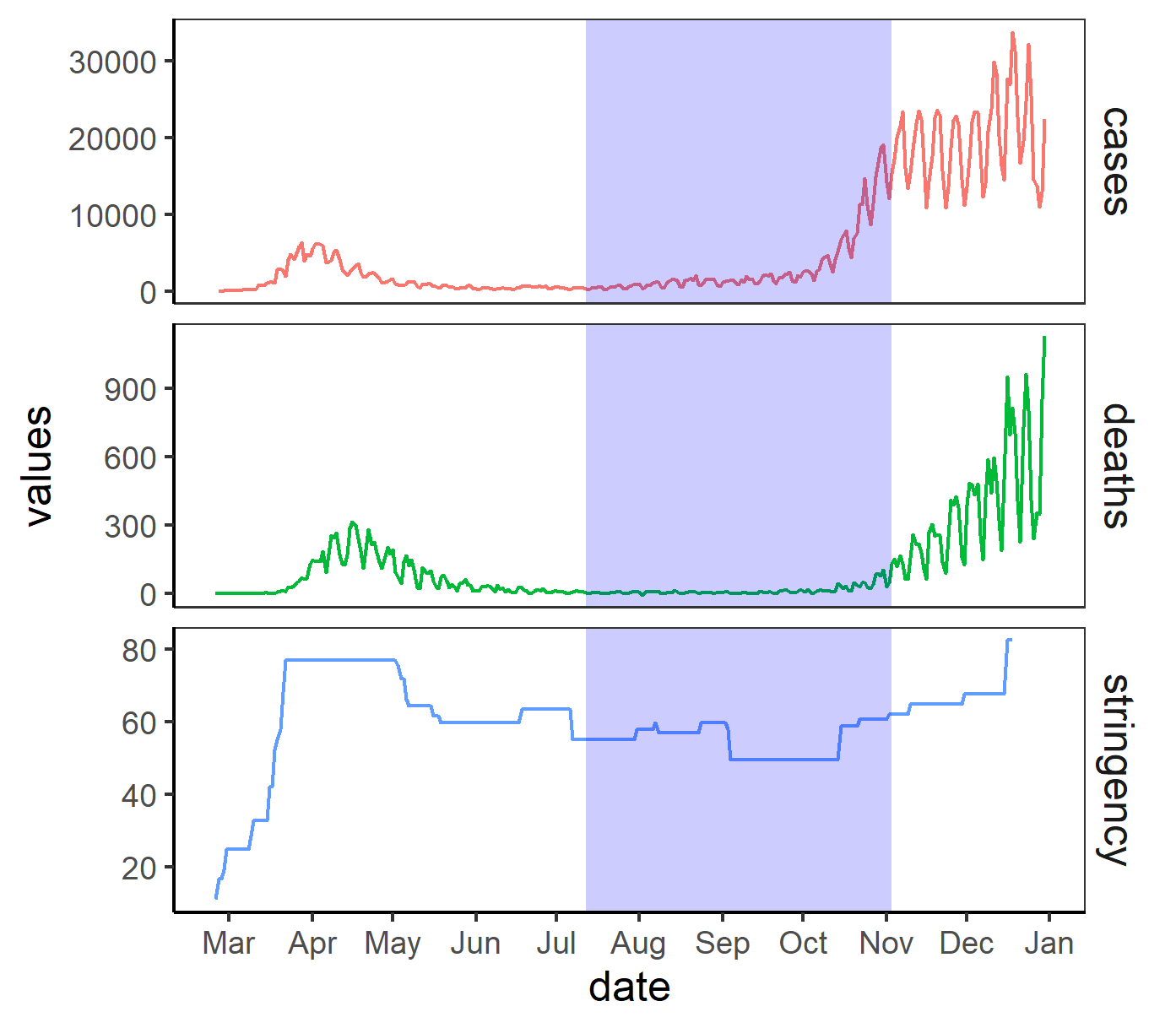


*Note.* “cases” and “deaths” refer to daily COVID-19 incidence and death rates in Germany [1]. “stringency” displays an index of German government policies in response to the pandemic [2].

**References**

1. Robert Koch Institute. Coronavirus Disease 2019 (COVID-19), daily situation report of the Robert Koch Institute. https://www.rki.de/DE/Content/InfAZ/N/Neuartiges_Coronavirus/Situationsberichte/Jan_2021/2021-01-17-en.pdf?__blob=publicationFile/. Accessed March 18, 2021.

2. Our World in Data. COVID-19: Government Stringency Index. https://ourworldindata.org/grapher/covid-stringency-index/. Accessed March 18, 2021.
