# Supplementary material for: Hand Washing and Related Cognitions Following a Brief Behavior Change Intervention During the COVID-19 Pandemic: a Pre-Post Analysis
Source: Int J Behav Med. 2021 Nov 29;29(5):575–86. doi: 10.1007/s12529-021-10042-w (PMC8628490; doi:10.1007/s12529-021-10042-w)

Electronic Supplementary Material 2:
Study material

A: Registration
B: Intervention at baseline (Day 0)

[after persons saw the study advertisement
(on the right; in German) and clicked on the study link]

A: Registration

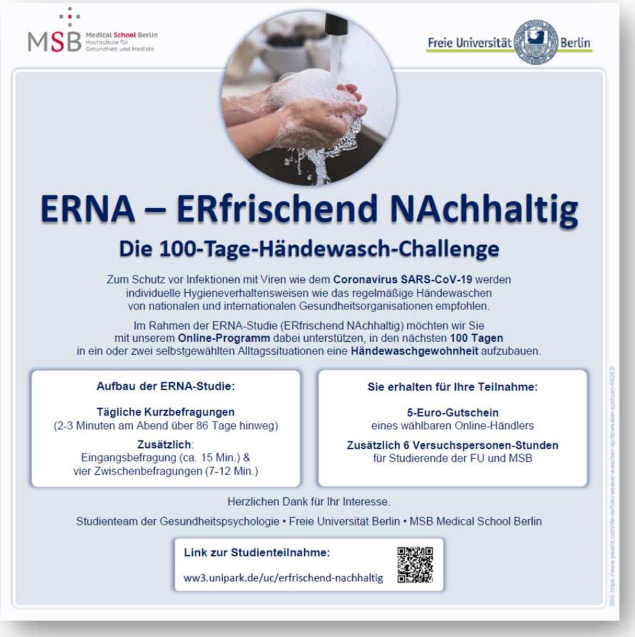

Welcome!

A common preconception is that it takes 3 weeks to form a **new habit**. However, research* suggests that this can vary from person to person and it takes more than 3 weeks.

To protect against viral infections such as SARS-CoV-19 (coronavirus), national and international health organizations (Robert Koch Institute; World Health Organization, WHO) recommend to regularly **wash hands**.

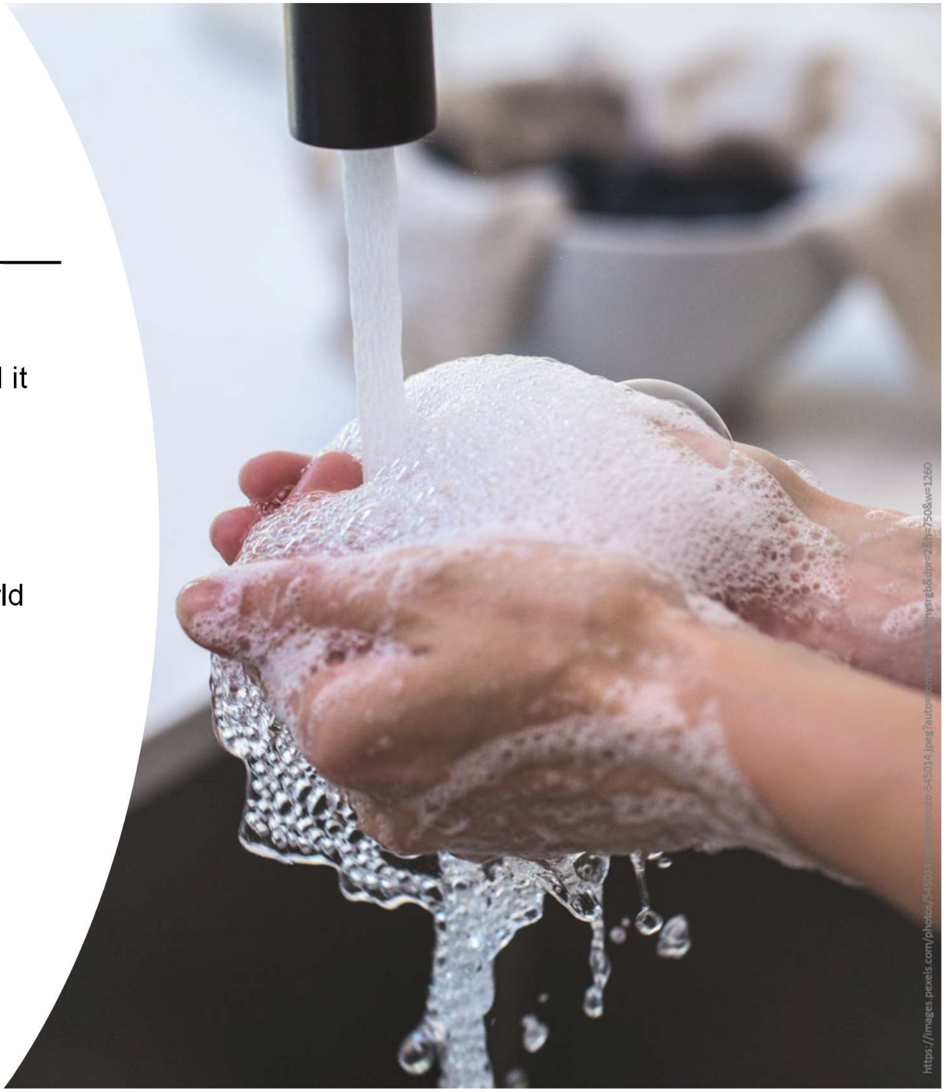

* Lally, P., Van Jaarsveld, C. H., Potts, H. W., & Wardle, J. (2010). How are habits formed: Modelling habit formation in the real world. *European Journal of Social Psychology*, 40(6), 998-1009.

A New Hand Washing Habit

When hand washing becomes a habit in certain everyday situations, you no longer have to think about it, it makes your **everyday life much easier**.

While some everyday situations automatically trigger hand washing, such as washing hands after using the toilet, there may also be **situations** in which you want to develop a **new hand washing habit.**

In the **ERNA study** (German: „ERfrischend NAchhaltig"), we would like to support you creating a **new hand washing habit** in up to two everyday situations of your choice over the next **100 days.**

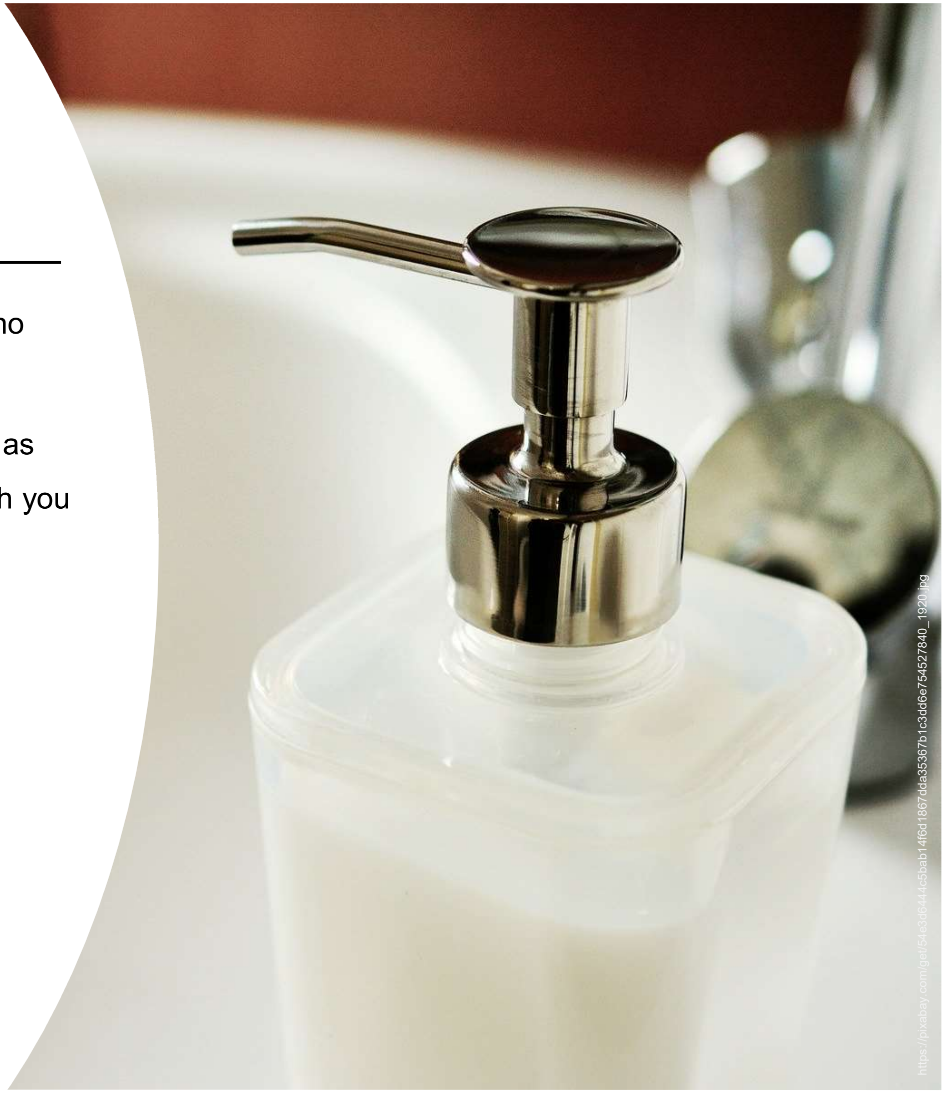

ERNA – The Online Program

ERNA is an **online program** developed by health psychologists from the - *blinded for review* - and the - *blinded for review.*

We will ask you to fill in a short daily questionnaires (2-3 minutes for 86 days), there will be an initial survey (15 minutes) and four other surveys later on (7-12 minutes).

By participating, you support psychological research on habit formation.

Furthermore, after completing the study, you will receive a **5 EUR voucher** from an online shop.

Additionally, participating students from - *the blinded for review* - and the - *blinded for review* – will receive **6 course credits.**

Are you ready for ERNA? Then please click on "Next" and confirm your participation after reading the study information sheet.

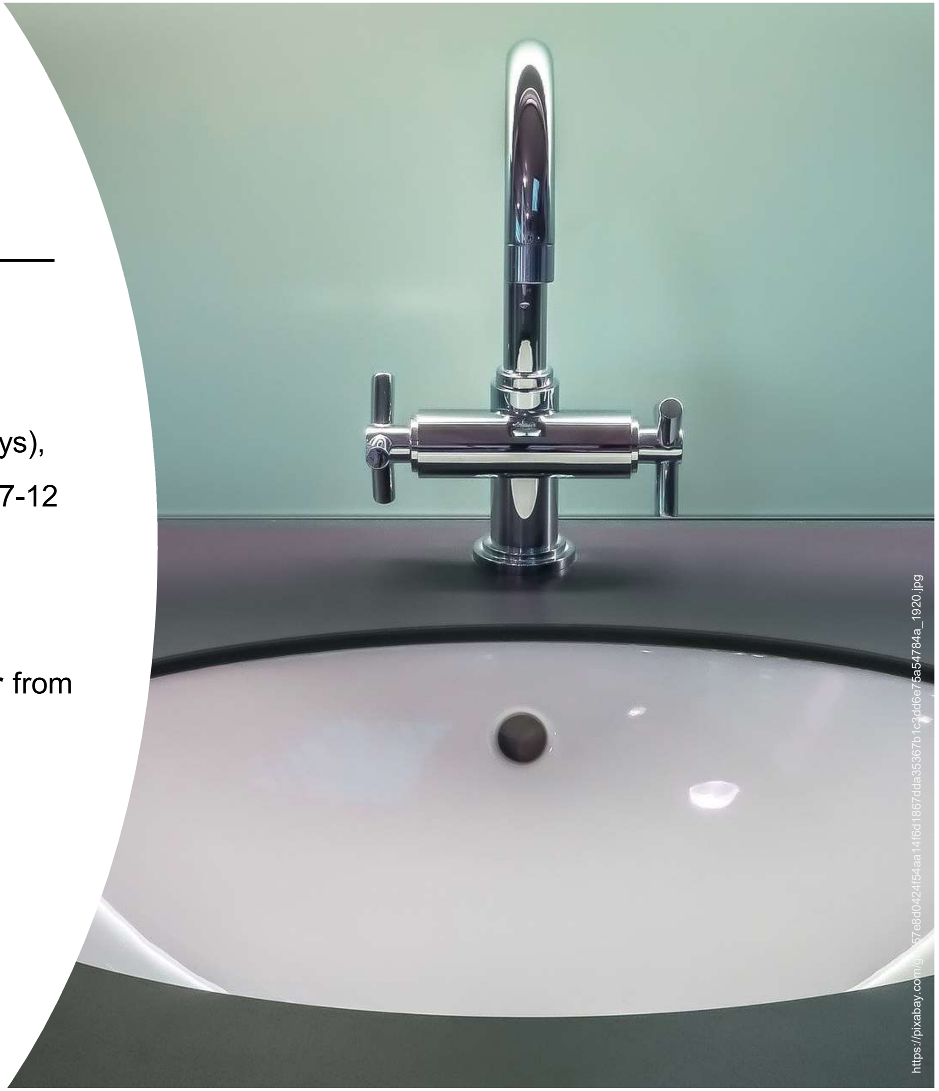

[informed consent & email contact]

Further Information

Thank you for entering your email address!

On the following pages you will find information about research on **habit formation** and your **100-days hand washing challenge**, which will begin after the next online session.

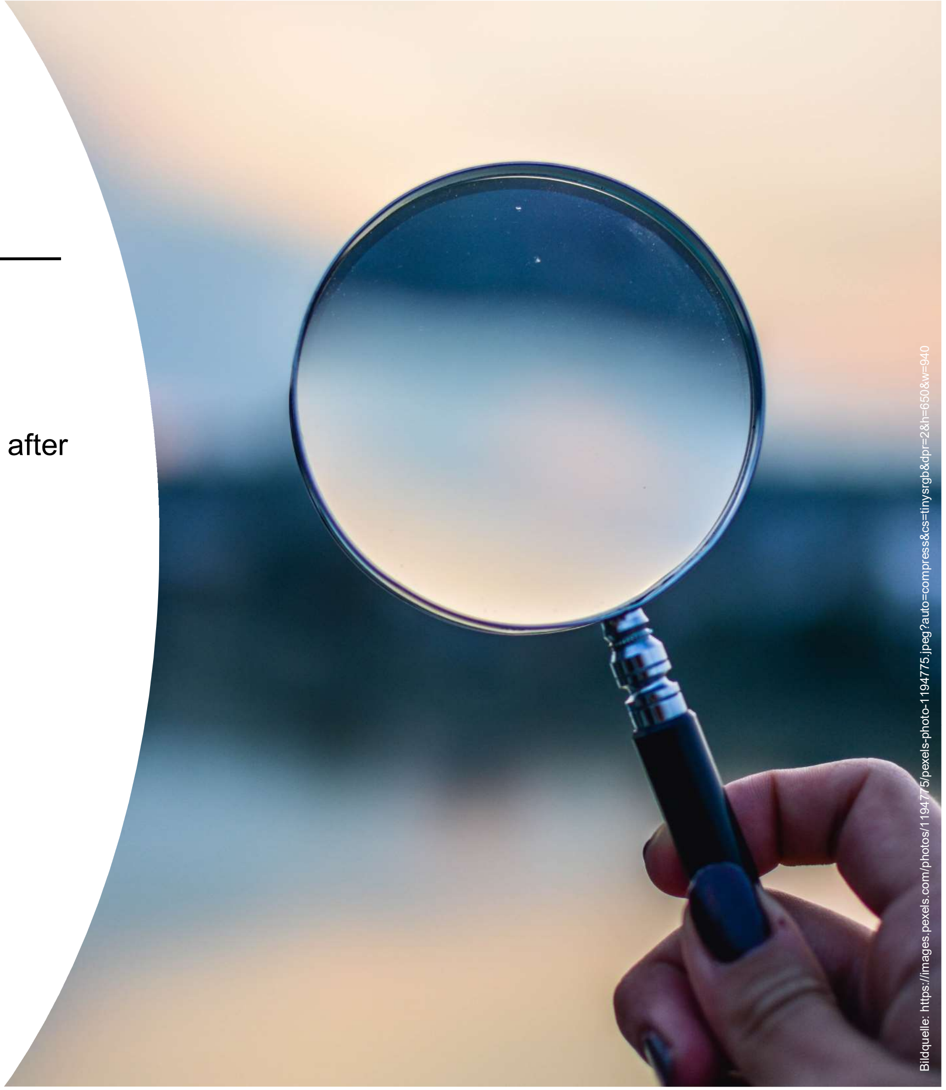

Bildquelle: https://images.pexels.com/photos/1194775/pexels-photo-1194775.jpeg?auto=compress&cs=tinysrgb&dpr=2&h=650&w=940

Your 100-Days Hand Washing Challenge

Before filling in the first questionnaire, please think of **one or two everyday situations** in which you would like to develop a new **hand washing habit** and in which hand washing is not already habitual.

Such an everyday situation is your "**personal trigger**".

In the next few days you will receive an **email** with the invitation about the next **online session**.

During the 100 days after your next online session, the challenge is to **wash your hands** whenever your **personal trigger** occurs.

Then, **your 100-days hand washing challenge begins**.

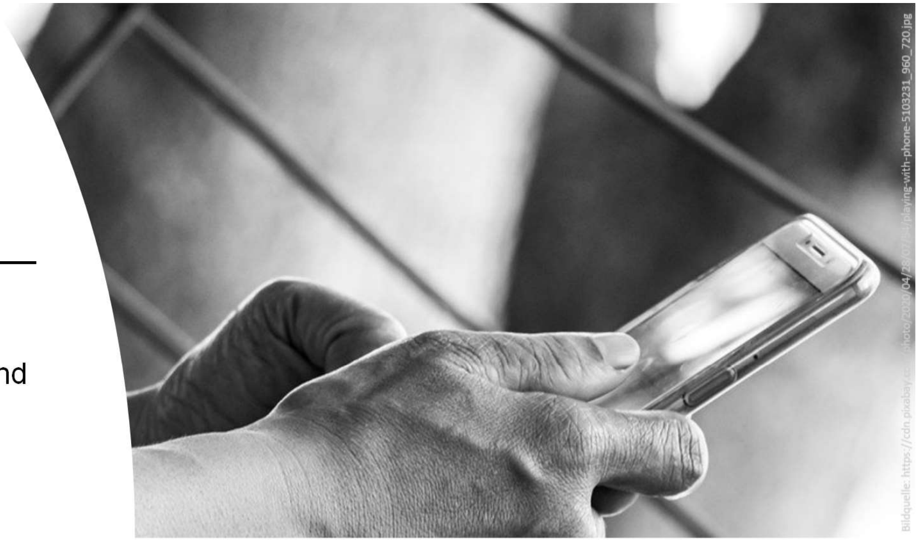

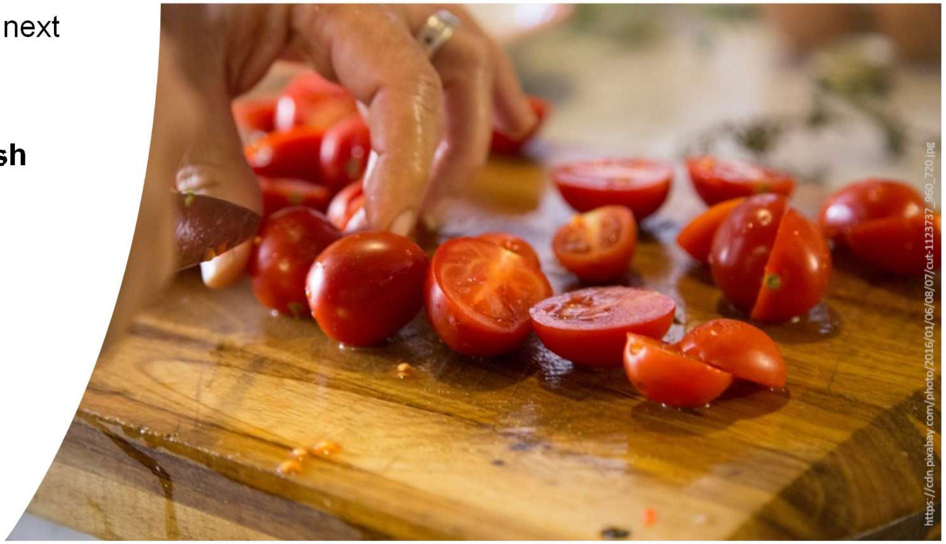

Personal Triggers Will Help You Build New Habits

Habits are behaviors that happen without you having to think about them.

Applied research has shown that it is easier to form habits when you associate the **desired behavior with certain everyday situations.** These can be locations or existing routines.

The key to successful habit formation is to carry out the behavior when this **personal trigger or everyday situation** occurs.

Initially, this requires memory and willpower. However, this reduces over time and, at some point in time, the personal trigger alone is enough to automatically lead to the new behavior: **A new habit has been developed**.

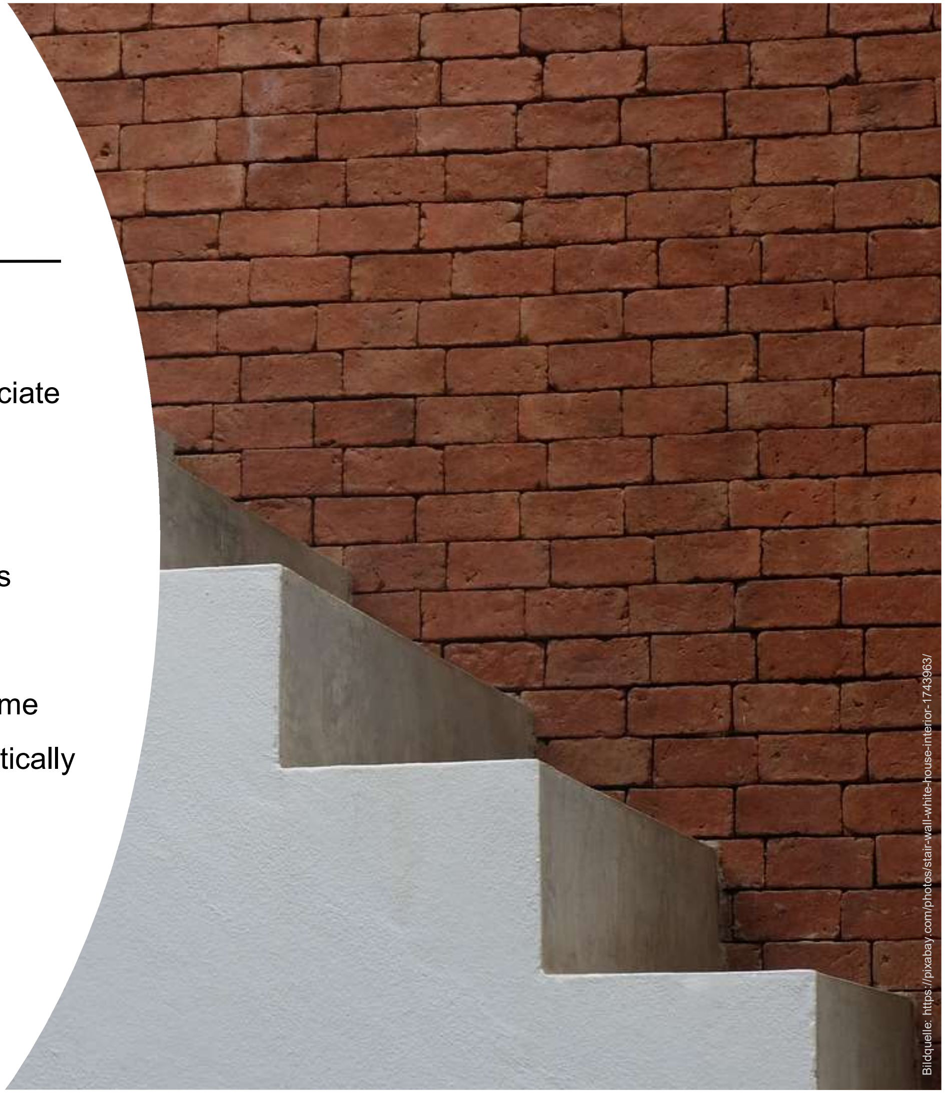

Situations in Which Hand Washing is Crucial

When your hands are dirty, you usually wash them automatically. But pathogens which cannot be seen with the naked eye can also be found on clean hands.

Therefore, the Federal Center for Health Education recommends to pay particular attention to hand washing when the following situations occur (among others):

- After blowing your **nose, coughing** or **sneezing**
- After contact with **rubbish**
- Before **meals**
- Before handling **medication** or **cosmetics**

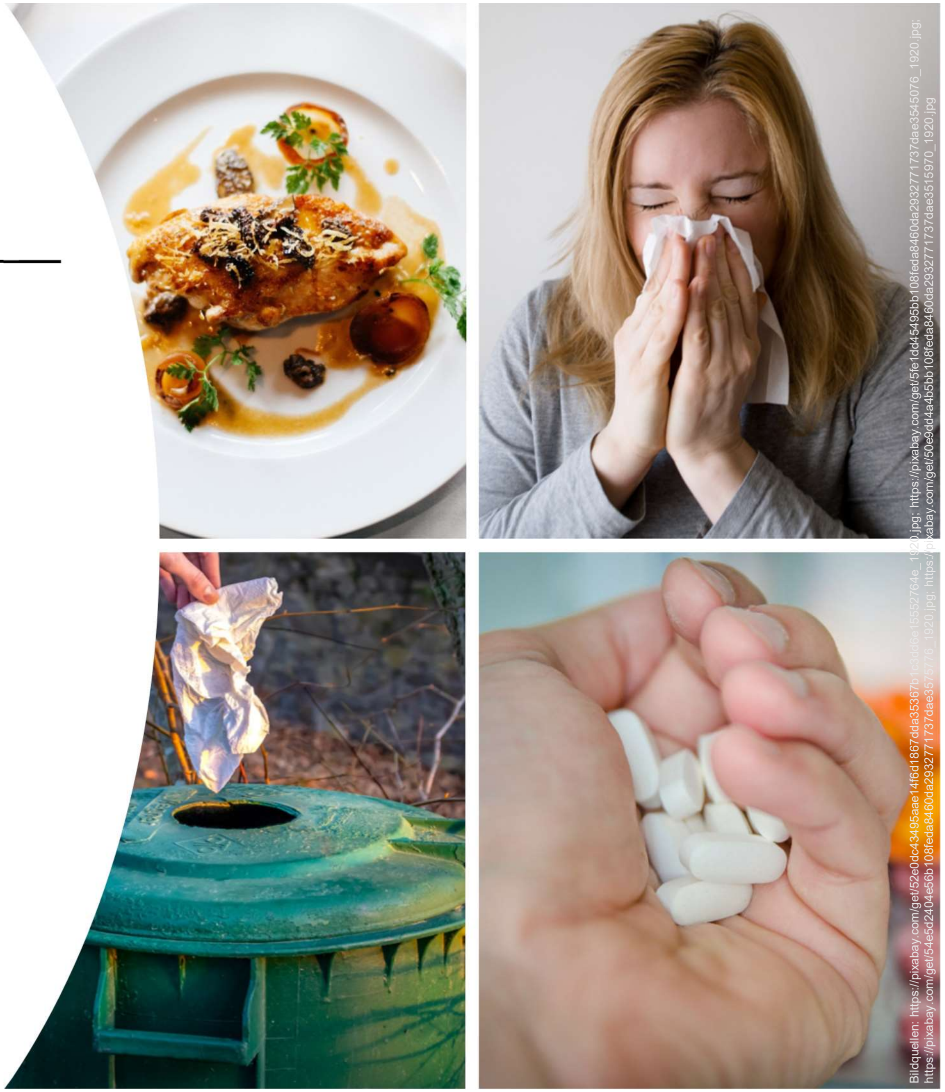

Bildquellen: https://pixabay.com/get/52e0dc43495aae14f6d1867dda35367b1c3dd6e15552764e_1920.jpg; https://pixabay.com/get/5fe1dd45495bb108feda8460da293277 1737dae3545076_1920.jpg; https://pixabay.com/get/54e5d2404e56b108feda8460da293277 1737dae3575776_1920.jpg; https://pixabay.com/get/50e9dd4a4b5bb108feda8460da293277 1737dae3515970_1920.jpg

In Everyday Life, it can be a Challenge to Always Remember to Wash Hands

In which situations do you find it particularly difficult to remember to wash your hands?

Which situations are challenging for you?

Are there situations in which you have never or rarely washed your hands, but would like to do so more often in the future?

Examples of such daily life situations:

- After using the **cell phone** for a long time.
- When I arrive at **work**.
- Before I start **preparing a meal** at home.
- Before I start **snacking** at home.

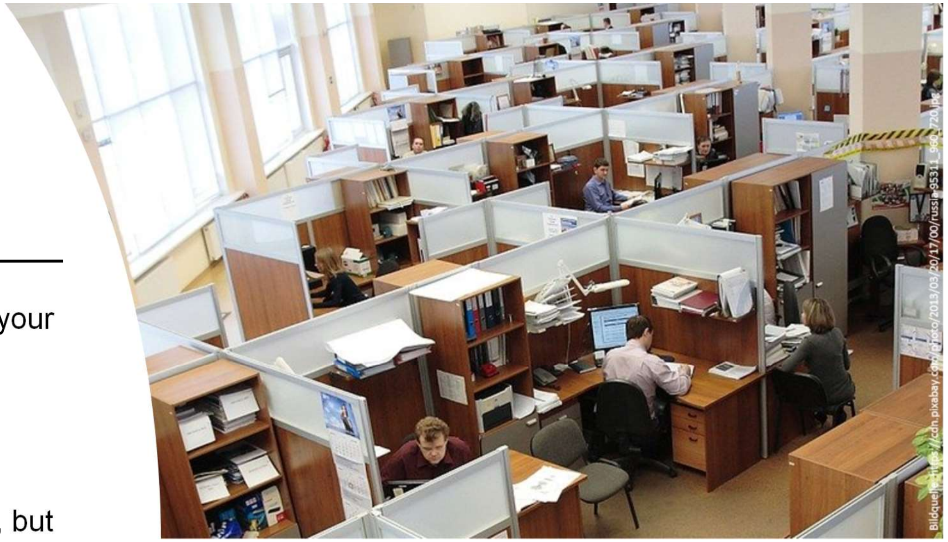

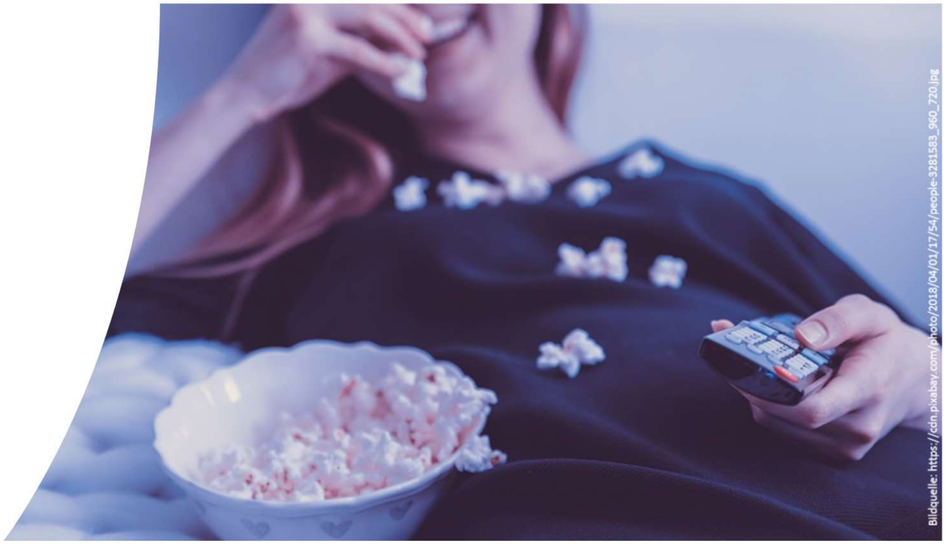

Thank you!

That‘s it for today.

We will email you **within the next few days** when it is time for your next online session and the selection of your everyday situations for your **100-days hand washing challenge**.

If you have any questions in the meantime, please email us at – blinded.

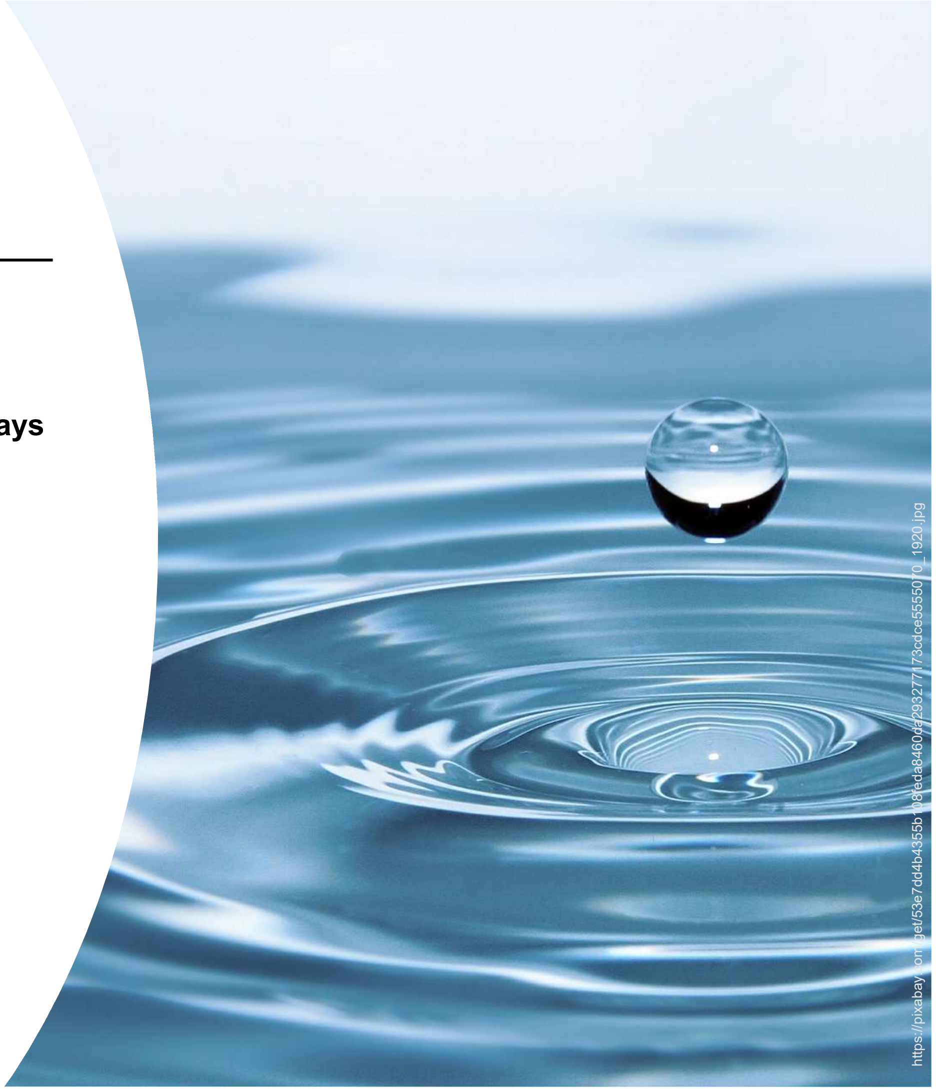

[a few days later]

B: Intervention at baseline (Day 0)

Your 100-Days Hand Washing Challenge

Thank you for completing the questionnaire!

Let‘s focus on your 100-days hand washing challenge.

You will be able to download the pages of today‘s session at the end.

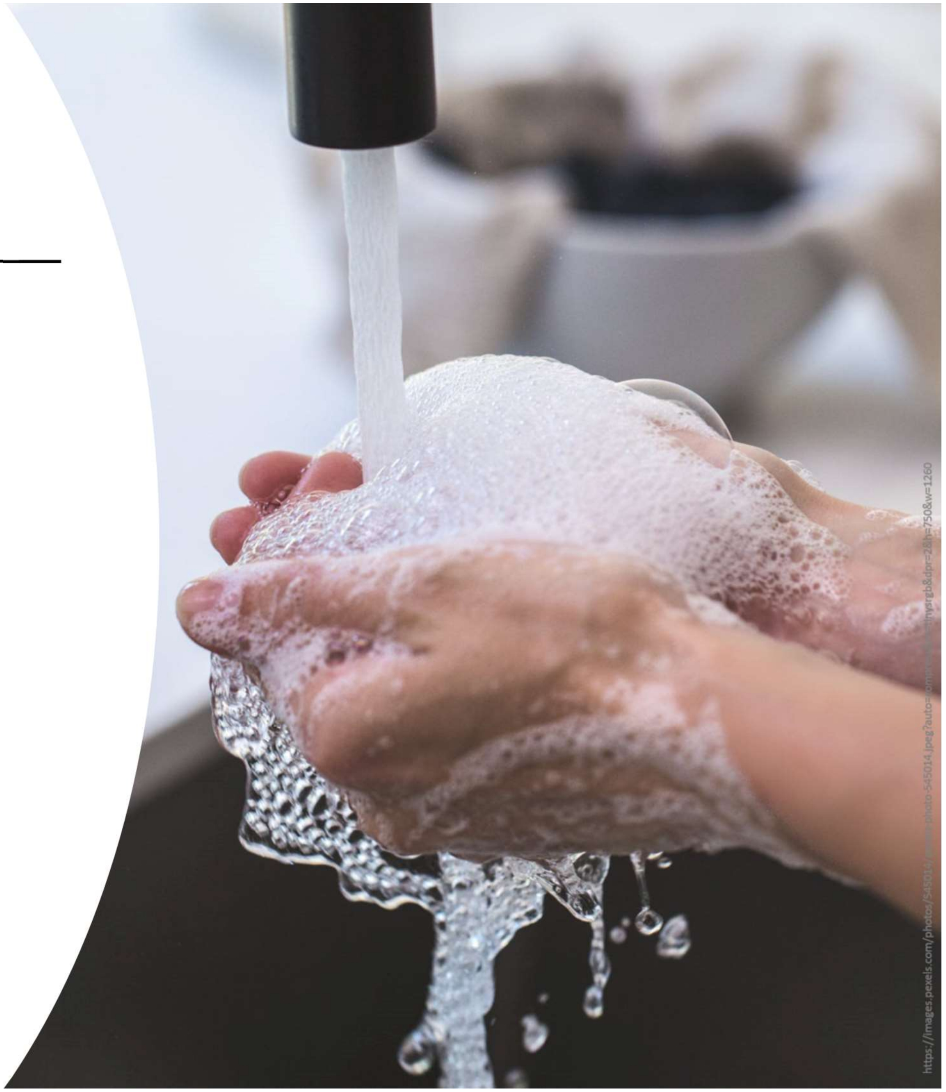

Small Actions with a Big Impact: Why Hand Washing is so Important?

Did you know that hands are main carriers of pathogens? Hand washing protects you against them.

When blowing your nose, going to the toilet, touching an animal or preparing raw meat: hands often come into contact with **germs** and can transfer them to anything that is subsequently touched.

When **shaking hands** or by **sharing objects**, pathogens can easily get from one hand to another.

If you then touch your **face** with your hands, the pathogens can enter the body via your mouth, nose or eyes and might trigger an infection.

Hand washing interrupts this transmission path.

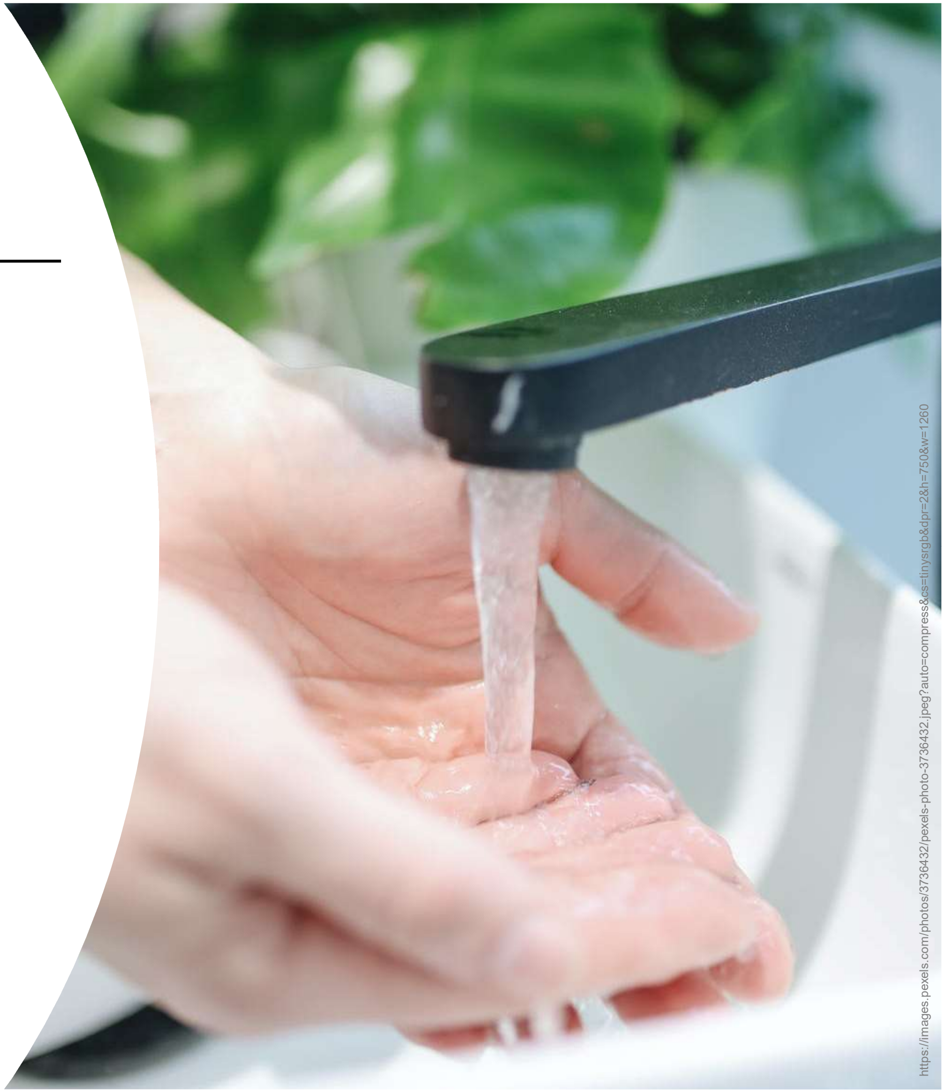

https://images.pexels.com/photos/3736432/pexels-photo-3736432.jpeg?auto=compress&cs=tinysrgb&dpr=2&h=750&w=1260

Small Actions with a Big Impact: Why Washing your Hands is so Important

As another tip: You are on a walk and there is **no washing facility** and **no sanitizer** available?

Try to keep your hands **below your shoulders** and take the next hand washing opportunity that suits you.

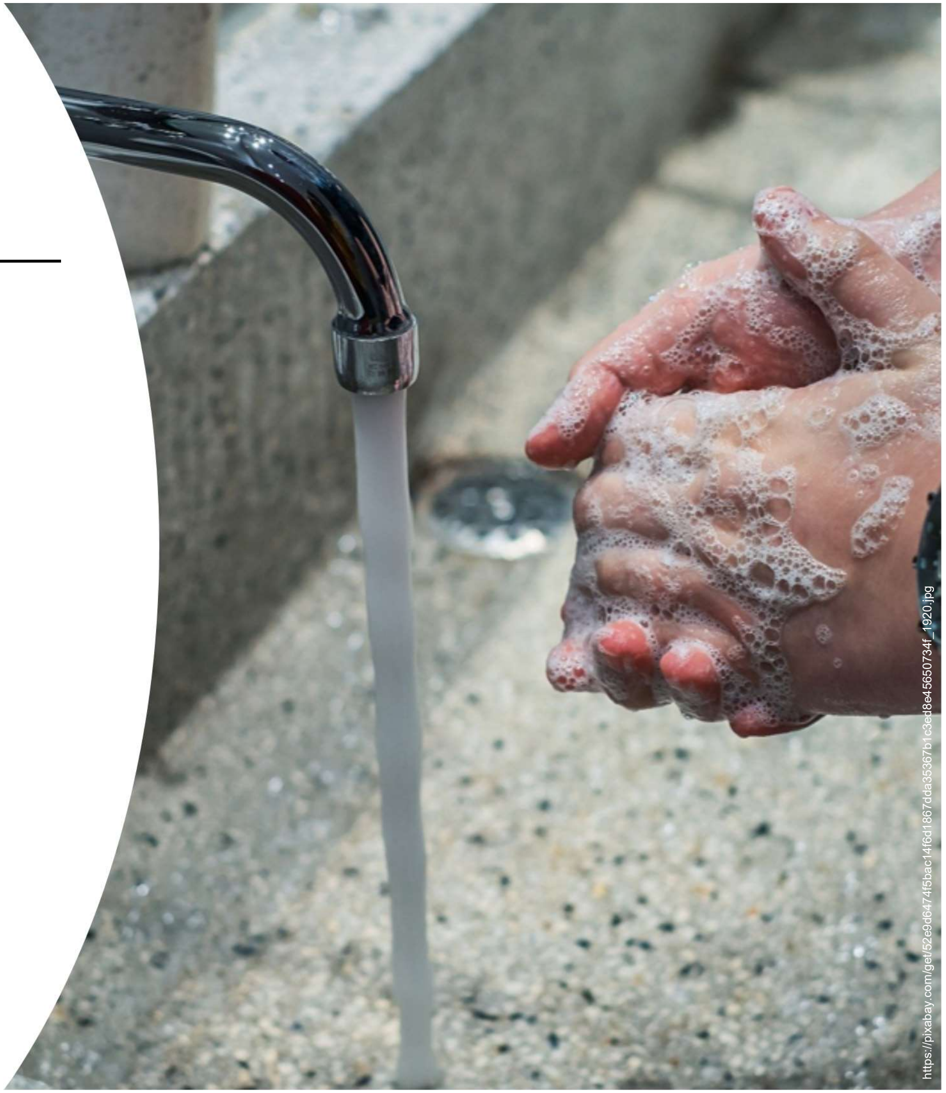

Hand Washing Protects Against Infections

Pathogens for many **infectious diseases** are transmitted by hands. These include illnesses such as colds, flu, or COVID-19.

Hand washing is a simple and effective measure that can **protect** you and others from **infection**.

If you **thoroughly** wash your hands on a **regular** basis, you can protect yourself and others from many pathogens.

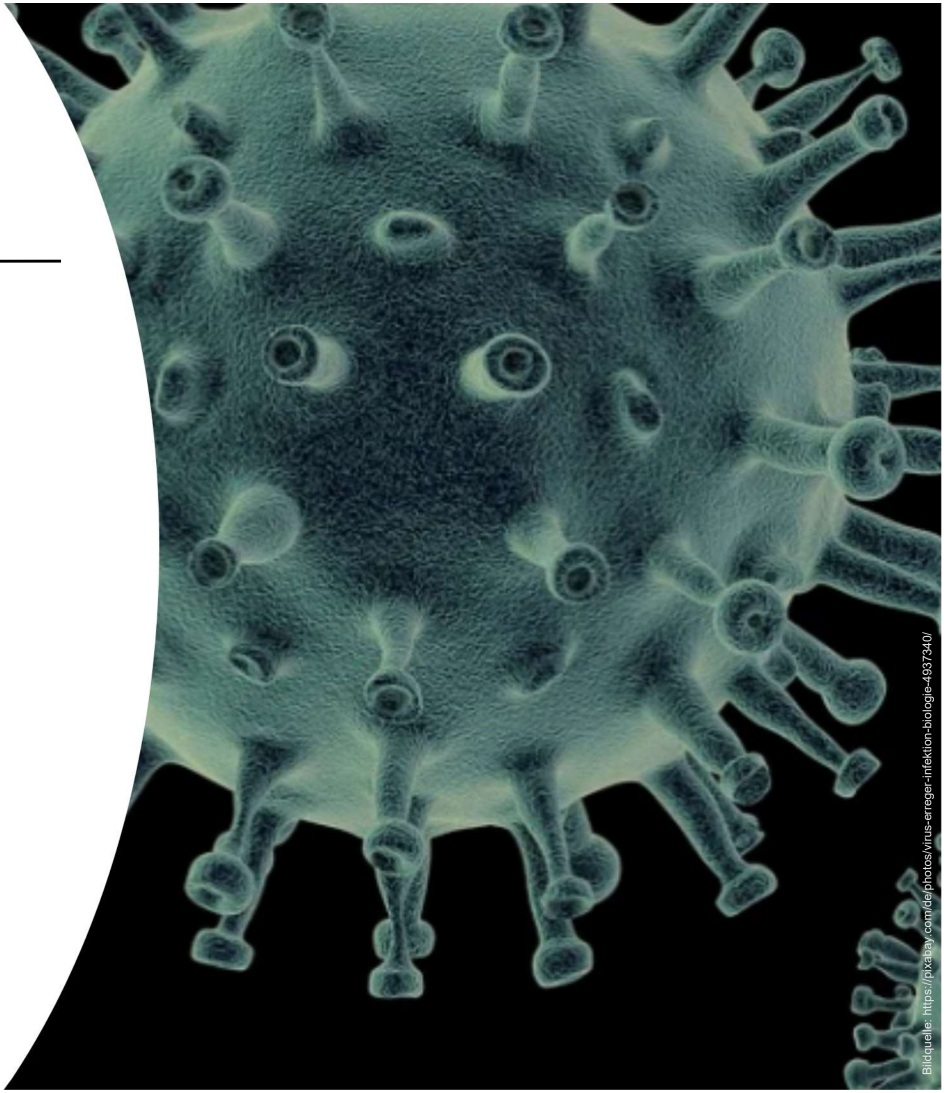

Reference: https://www.infektionsschutz.de/haendewaschen/#c6352

Washing Hands Protects Against Infections

Hand washing **reduces the number of germs** on the hands to up to a **thousandth**.

Soap and cleaning agents damage the shell of viruses - the **pathogens are inactivated**, even if only cold water is available.

This reduces the risk of pathogens entering the body, for example through the **mouth**, **nose** or **eyes**, or being passed on to other people.

This is particularly important in times of epidemics or pandemics.

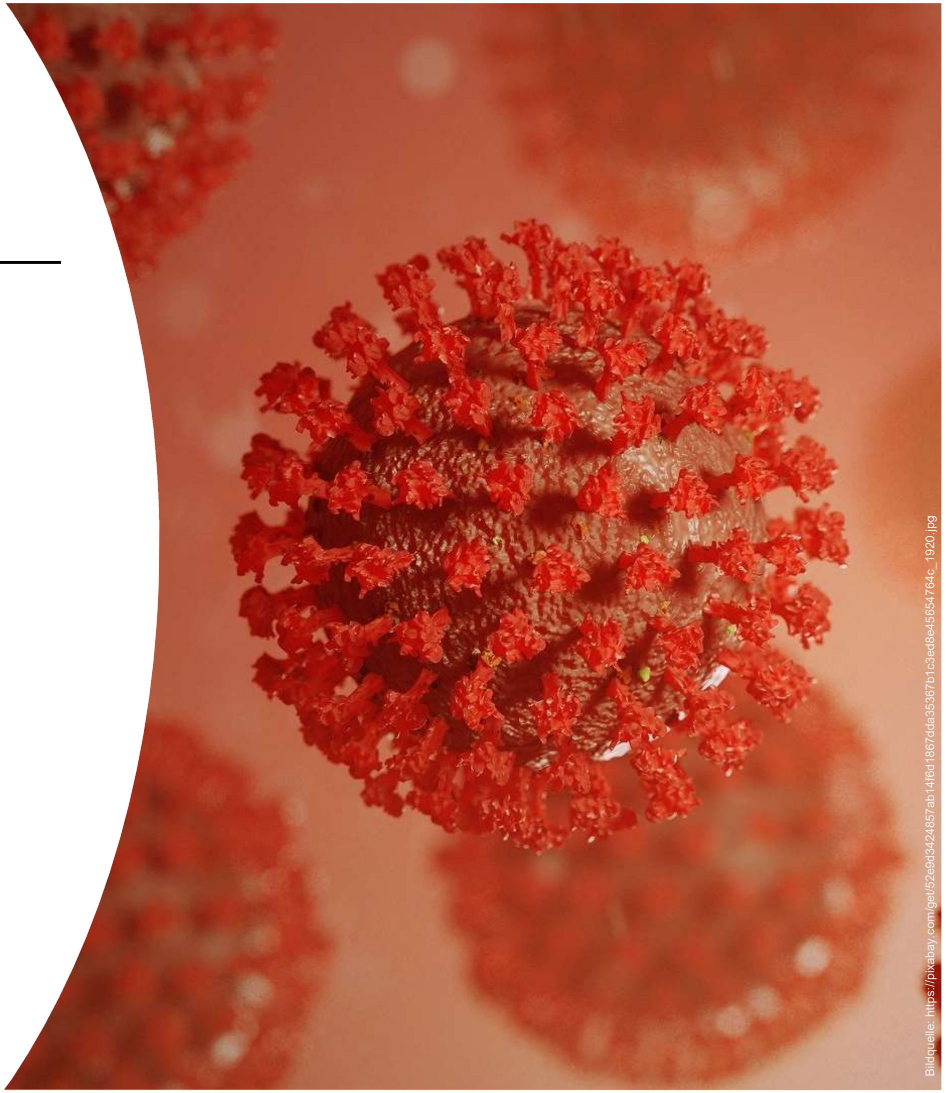

Reference: https://www.infektionsschutz.de/haendewaschen/#c6352

High-5 for Effective Hand Washing - Do You Know All 5 Tricks?

Step 1:

First, hold your hands under **running water**.

You should choose the **temperature** so that it is **comfortable**.

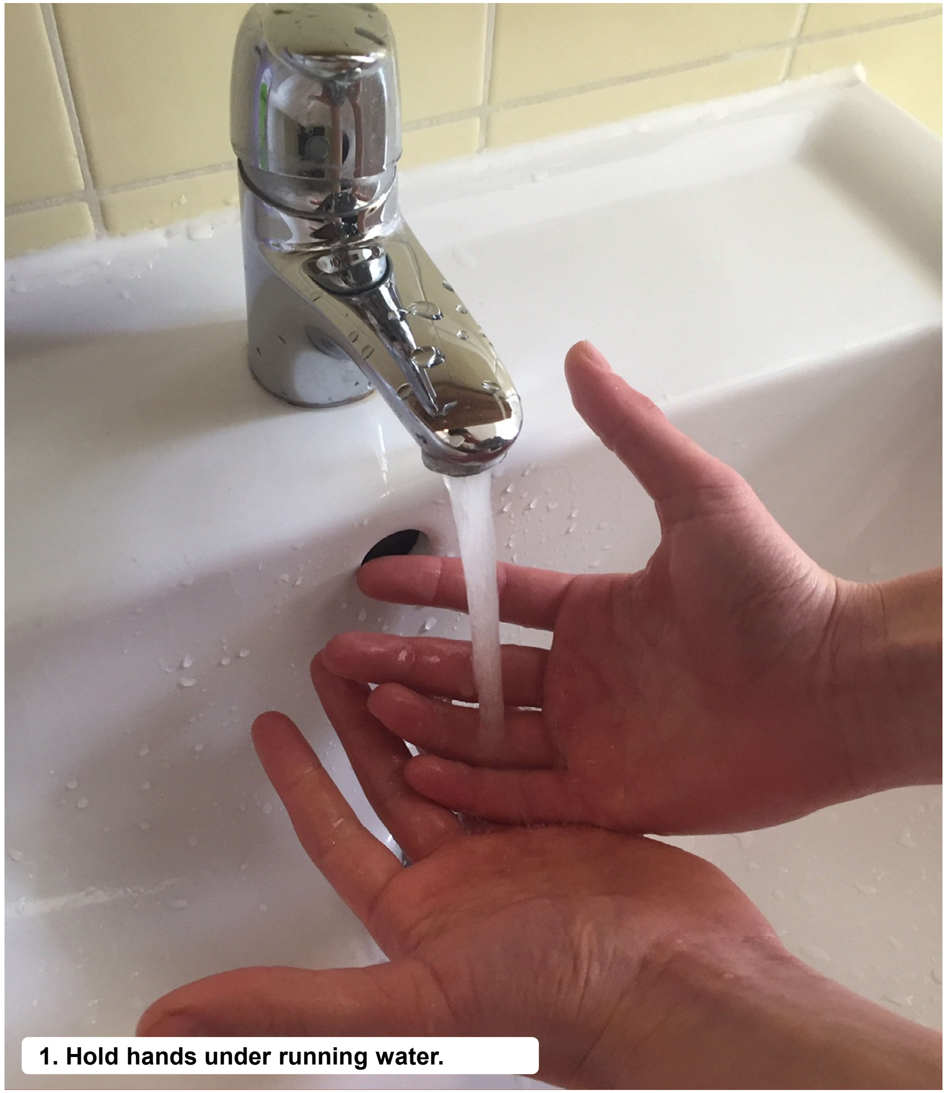

1. Hold hands under running water.

High-5 for Effective Hand Washing - Does your Soap Get on all Surfaces of your Hand?

Step 2:

Soap your hands thoroughly (2a)

and wash the following surfaces:

2b. Palms

2c. Back of the hands

2d. Fingertips

2e. Between the fingers

2f. Thumbs

2g. Fingernails

2h. Wrists

Liquid soap is better than hard soap, especially in public washrooms.

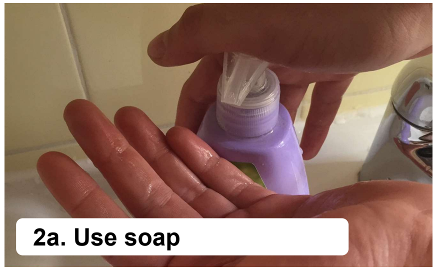

2a. Use soap

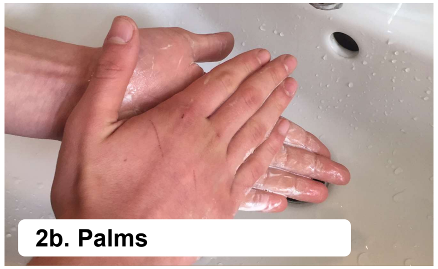

2b. Palms

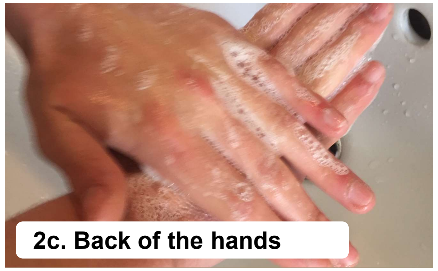

2c. Back of the hands

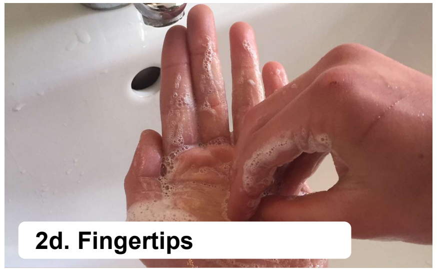

2d. Fingertips

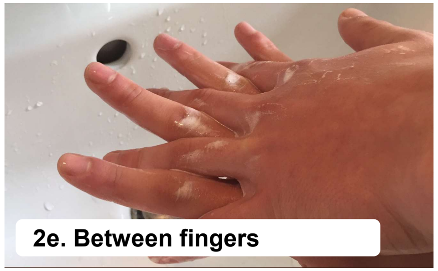

2e. Between fingers

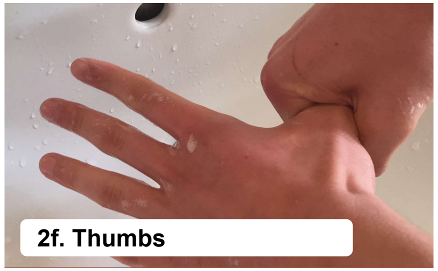

2f. Thumbs

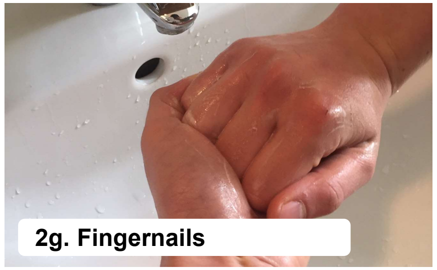

2g. Fingernails

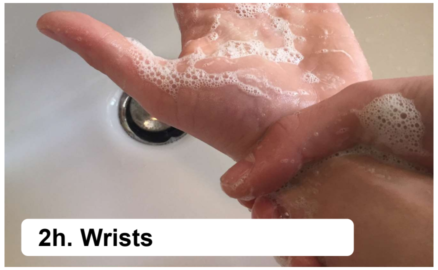

2h. Wrists

High-5 for Effective Hand Washing – How long do you Wash your Hands?

Step 3:

Effective hand washing takes **20 to 30 seconds.**

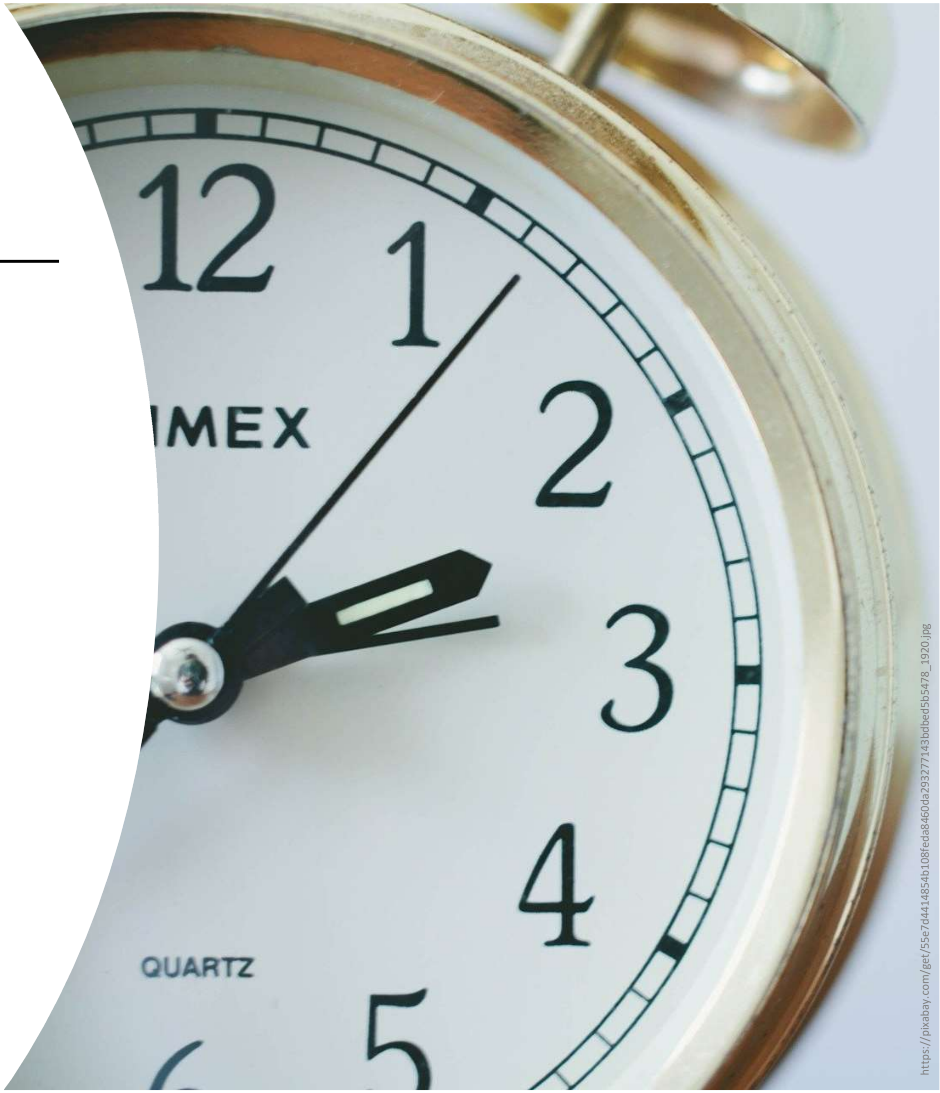

https://pixabay.com/get/55e7d4414854b108feda8460da293277143bdbed5b5478_1920.jpg

High-5 for Effective Hand Washing - The Elbow trick.

Step 4:

Rinse your hands under running water (4a).

Use a **disposable towel** or your **elbow** for the tap (4b), especially in public toilets.

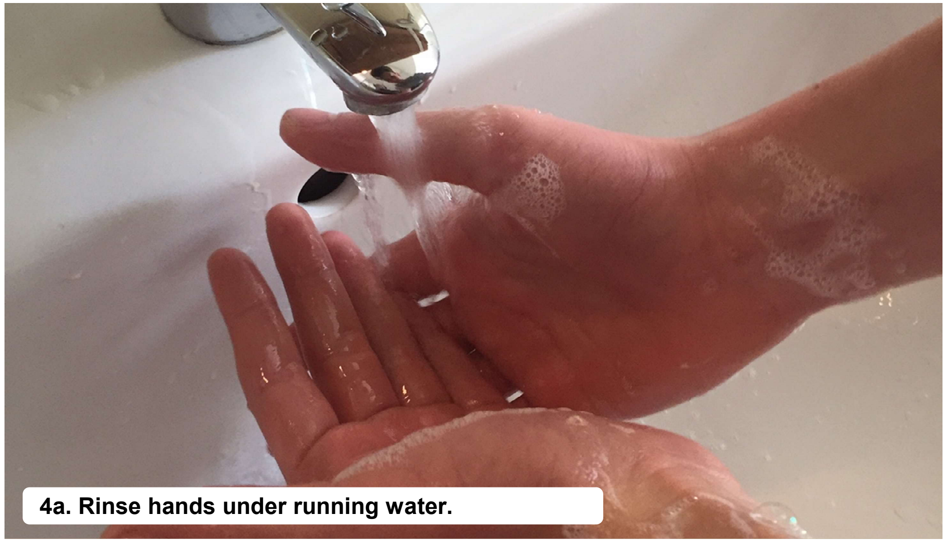

4a. Rinse hands under running water.

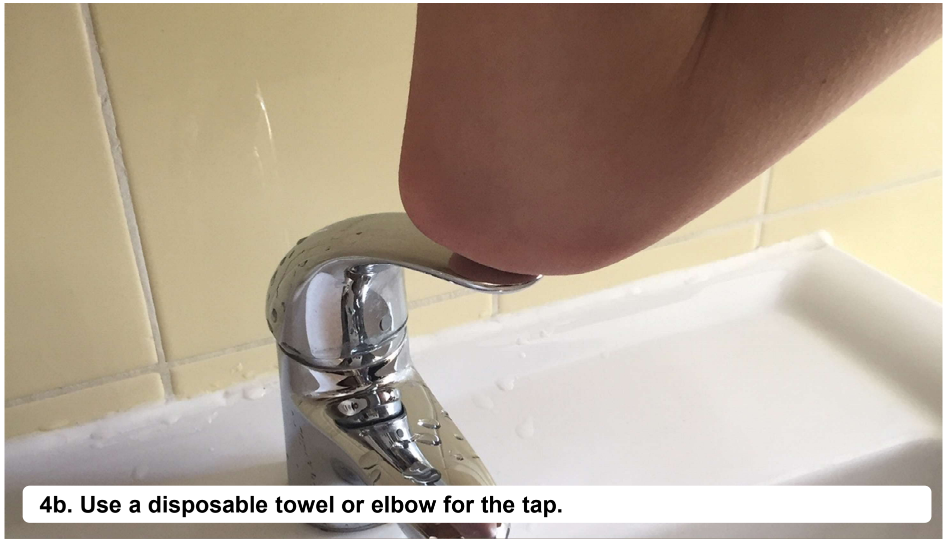

4b. Use a disposable towel or elbow for the tap.

High-5 for Effective Hand Washing – Do not Forget to Dry Your Hands ☺

Step 5:

In the end, dry your hands carefully, including the area between your fingers.

In public toilets, a **disposable towel** can be used.

At home, your **personal towel** works best.

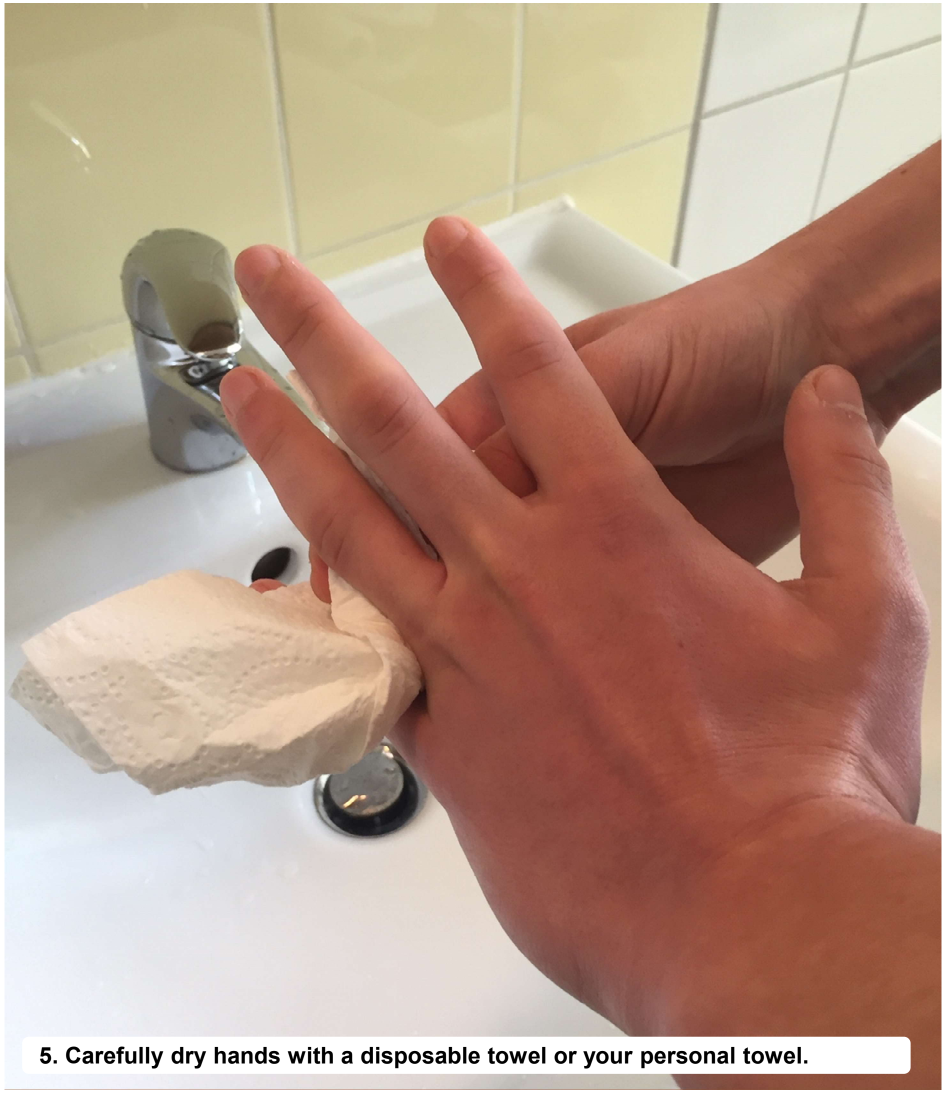

5. Carefully dry hands with a disposable towel or your personal towel.

What Motivates you to Wash your Hands?

Why do you wash your hands regularly? What are your main reasons?

My 1. reason: ______________________	My 2. reason: _______________________

Is there a moment or ritual when you particularly enjoy your hand washing?

A hand washing moment that I find pleasant or enjoy: ____________________________

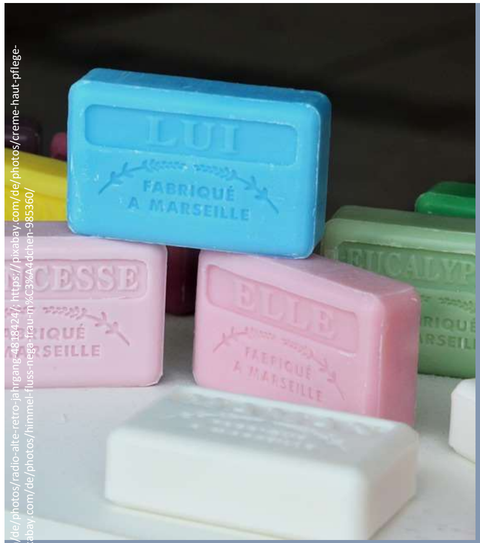

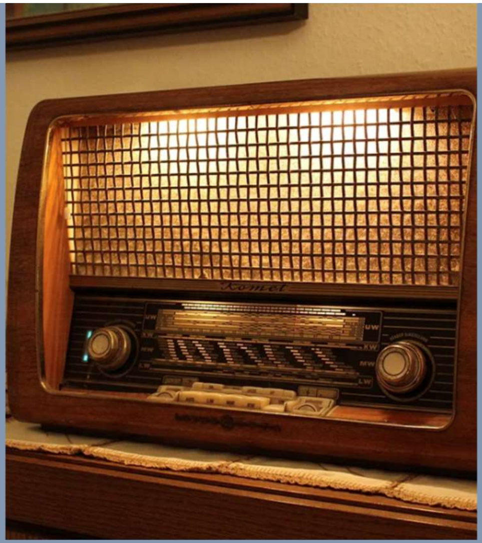

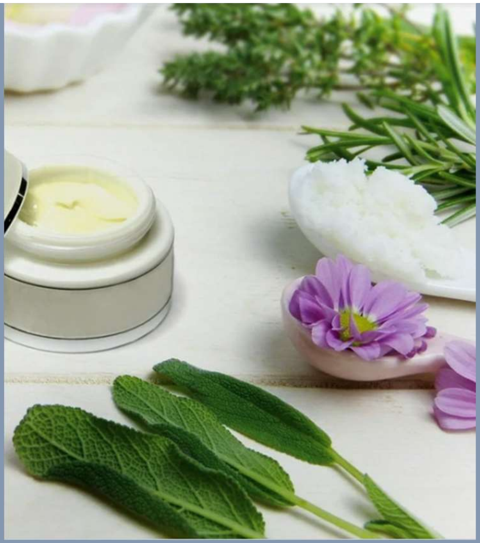

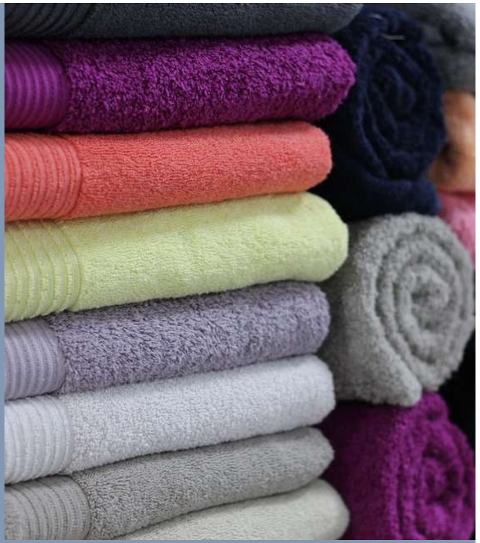

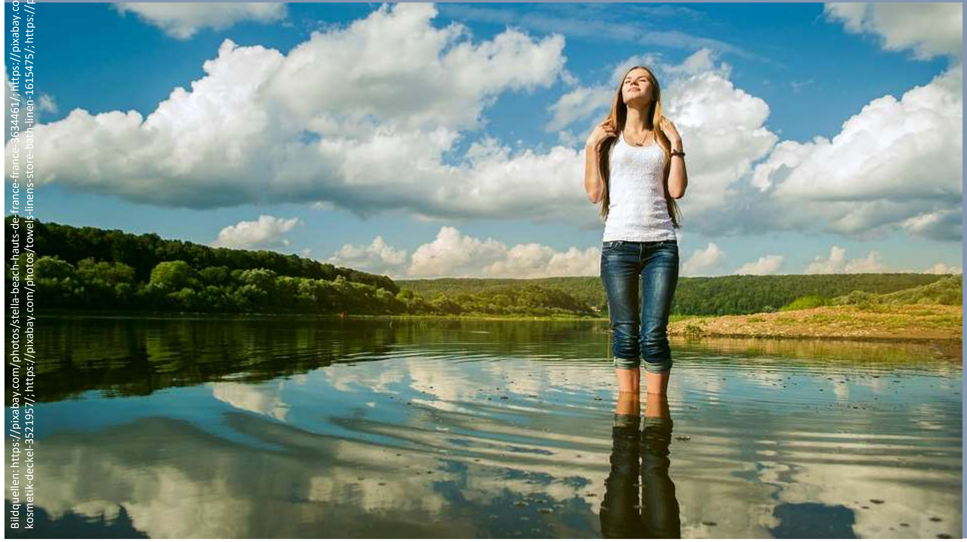

Hand Washing with a Twist:

- More hand washing as an opportunity to buy yourself a nice soap or a nice hand cream?
- Hand washing as a short break to sing your favorite song again (to fill the 20 seconds)?
- Hand washing to enjoy a comfortable temperature of the water?
- Hand washing to identify the smell of the soap?

Bildquellen: https://pixabay.com/photos/stella-beach-hauts-de-france-france-3634461/; https://pixabay.com/de/photos/radio-alte-retro-jahrgang-4818424/; https://pixabay.com/de/photos/creme-haut-pflege-kosmetik-deckel-3521957/; https://pixabay.com/photos/towels-linens-store-bath-linen-1615475/; https://pixabay.com/de/photos/himmel-fluss-nega-frau-m%C3%A4dchen-985360/

Your 100-Days Hand Washing Challenge

Please think about **one or two everyday situations** for which you would like to **develop a new hand washing habit**.

The challenge for the next **100 days** will be to **wash your hands** whenever these everyday situations occur.

Everyday situations can mean very different things that you can experience or observe in everyday life, such as:

- After using the **cell phone** for a long time
- When I arrive at **work**
- Before I start **preparing the meal** at home
- Before I start **snacking** at home

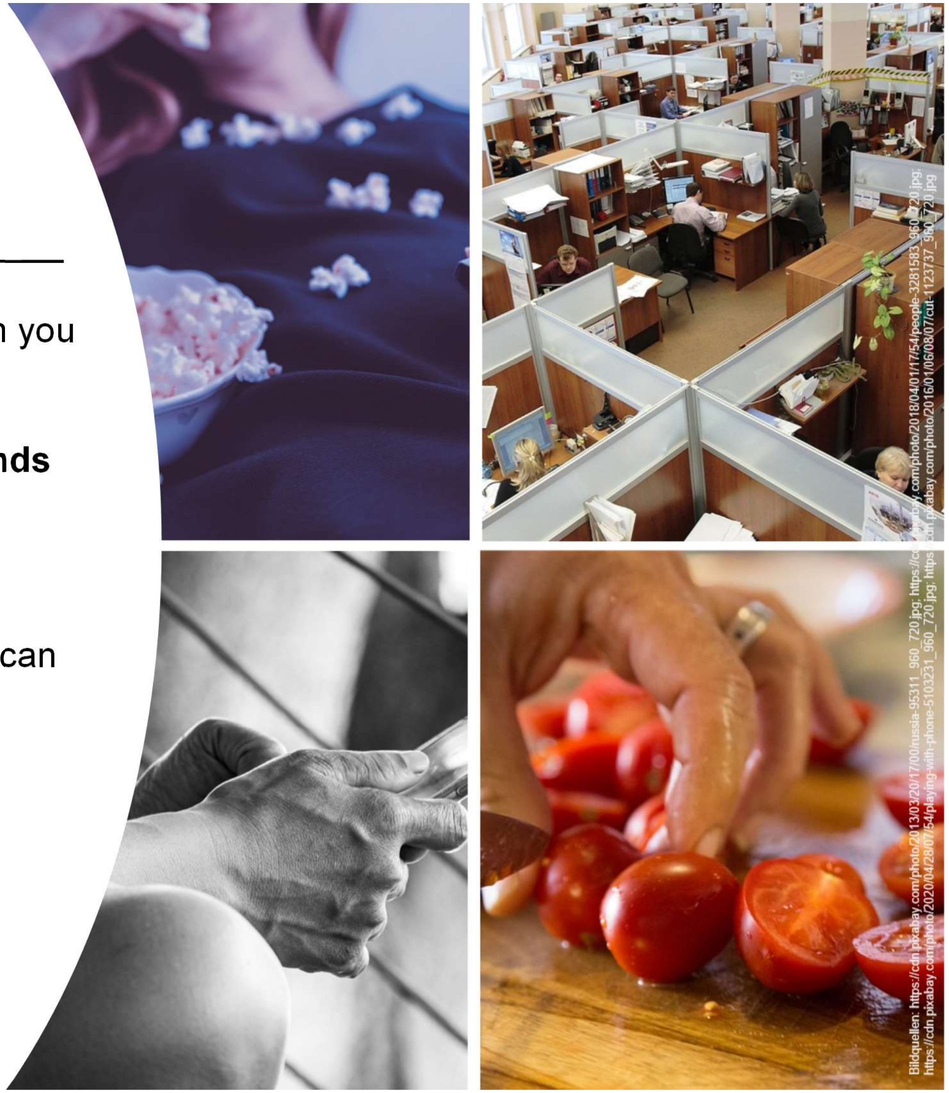

The 100-Days Hand Washing Challenge

Please enter up to 2 everyday situations in which you would like to develop a hand washing habit in the future.

Note:
At least one of the following criteria should apply to your everyday situation:

- In the past, I found it difficult to automatically wash my hands in this situation.
- In the past, I rarely or never washed my hands in this situation.

My everyday situation A:

My everyday situation B:

Important: Please make a note of your everyday situation(s) or take a photo or a screenshot of this page so that you can remember them and repeat them in the following questionnaires.

Thank you!

Thank you! You are done for today.

Your 100-days hand washing challenge will begin now – good luck! We will email you tomorrow evening when it is time for the first short questionnaire.

If you have any questions in the meantime, please email us.

As a thank you for your study participation, you will receive a 5 EUR voucher for an online shop at the end of the study as well as 6 course credits (if you are studying at the – blinded for review).

On the following page, you can also download information about your 100-days hand washing challenge.

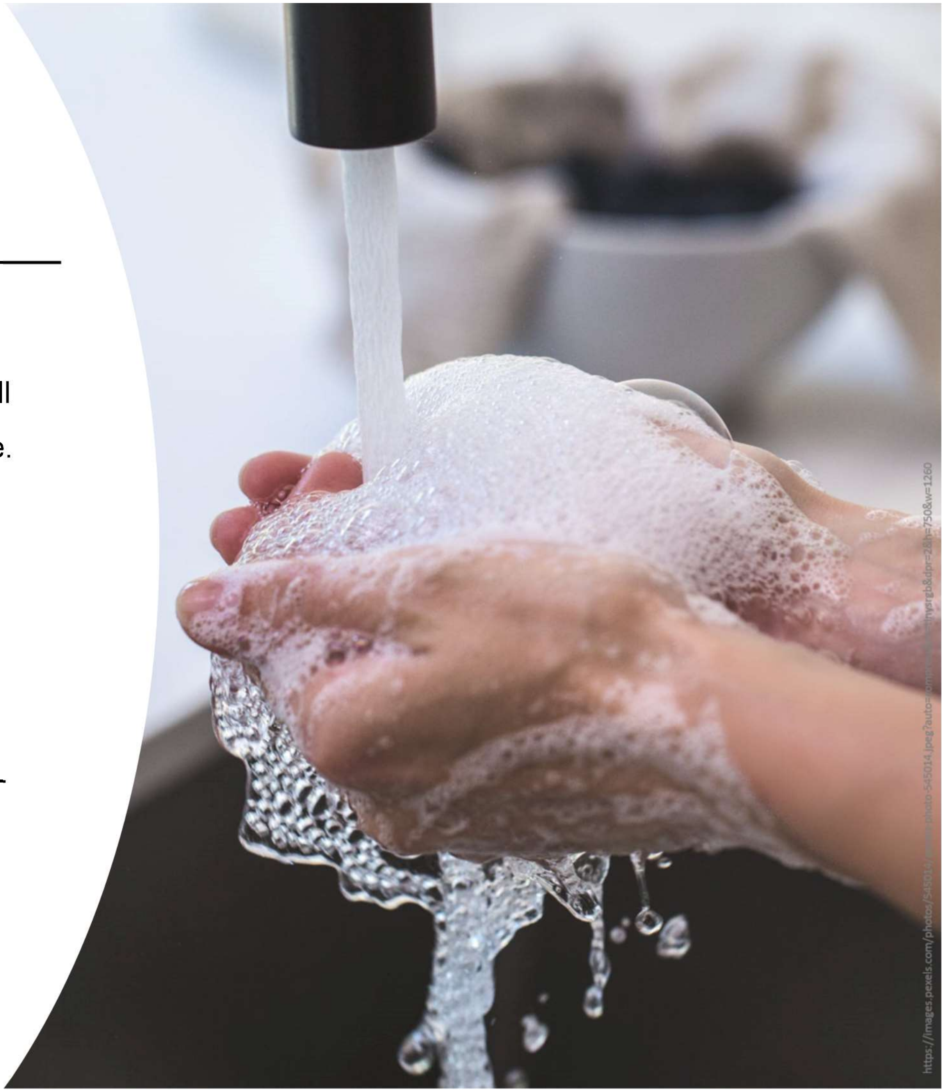

Supplement: Supplementary file 2 — Supplementary file2 (PDF 5274 KB) [file 12529_2021_10042_MOESM2_ESM.pdf]
