# Supplementary material for: Hand Washing and Related Cognitions Following a Brief Behavior Change Intervention During the COVID-19 Pandemic: a Pre-Post Analysis
Source: Int J Behav Med. 2021 Nov 29;29(5):575–86. doi: 10.1007/s12529-021-10042-w (PMC8628490; doi:10.1007/s12529-021-10042-w)
Supplement: Supplementary file 3 — Supplementary file3 (DOCX 72 KB) [file 12529_2021_10042_MOESM3_ESM.docx]

**Electronic Supplementary Material 3: Additional results**

Next to key study variables (intention, self-efficacy, and self-monitoring), further motivational and volitional HAPA variables were assessed. Using a scale from 1 to 6, positive outcome expectancies were assessed with the item “If I wash my hands regularly, then I would take care of my health”, negative outcome expectancies with the item “If I wash my hands regularly, then it would take too much of my time”, risk perception with the item “What is your risk of coronavirus SARS-CoV-2 infection, if you wash your hands regularly?”, and planning with the item “In the past 7 days, I have made a concrete plan regarding when and where to wash my hands for at least 20 seconds with soap and water”.

**
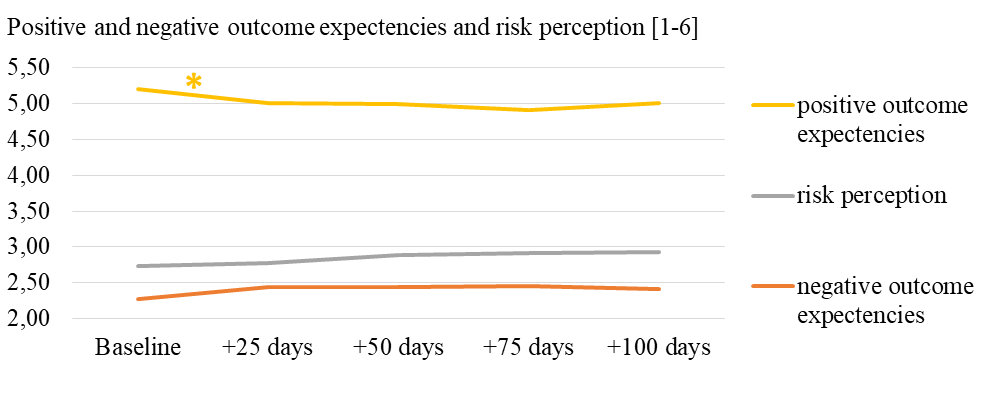
**

*Figure A.* Means of positive and negative outcome experiences and risk perception over time

*Note*. 68 ≤ *n ≤* 89 participants due to missing values. * *p* < .05. Significant between-assessment differences: positive outcome expectancies between D0 and D25 (decrease).

**
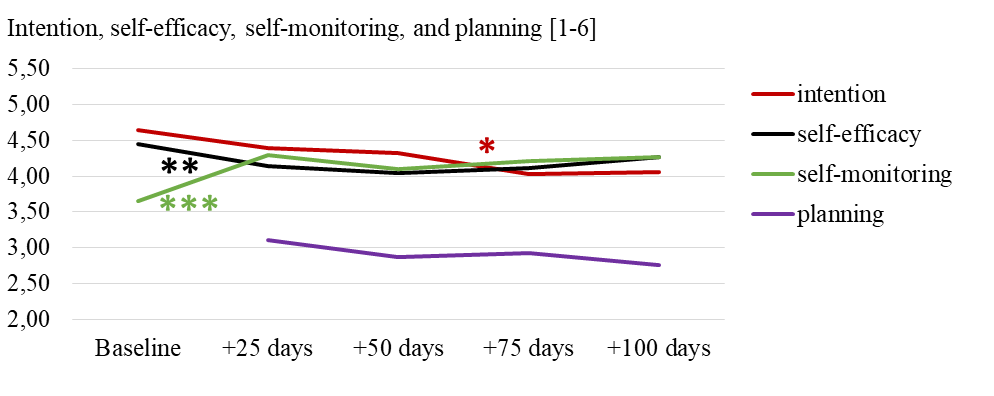
**

Figure B. Means of intention, self-efficacy, self-monitoring, and planning over time

*Note*. 67 ≤ *n ≤* 89 participants due to missing values. * *p <* .05; ***p <* .01; ****p <* .001. Significant between-assessment differences: intention between D50 and D75 (decrease); self-efficacy between D0 and D25 (decrease); self-monitoring between D0 and D25 (increase). Note that planning was not assessed at baseline due to technical issues.

Table A. *Fixed Effects Estimates for Two-level Models Predicting Hand Washing, With Participant-selected Hand Washing Situations as Additional Covariates*

| Outcome: Next-week hand washing  Fixed effects |  | |  |
| --- | --- | --- | --- |
|  | ***B* (*SE*)** | ***p*** | **95% CI** |
| Intercept (at study start, D0) | 5.02 (0.58) | <.001 | 3.87; 6.17 |
| Time (assessment following D0) | **0.45 (0.11)** | **<.001** | 0.22; 0.67 |
| Within- person intention | -0.09 (0.10) | .408 | -0.29; 0.12 |
| Between-person intention | 0.42 (0.40) | .299 | -0.38; 1.21 |
| Within-person self-efficacy | 0.21 (0.13) | .108 | -0.05; 0.47 |
| Between-person self-efficacy | -0.06 (0.43) | .890 | -0.80; 0.92 |
| Within-person self-monitoring | 0.16 (0.09) | .097 | -0.03; 0.34 |
| Between-person self-monitoring | **1.10 (0.41)** | **.009** | 0.28; 1.91 |
| Age | 0.11 (0.06) | .057 | -0.01; 0.22 |
| Sex (0 = female; 1 = male) | -0.69 (0.82) | .399 | -2.32; 0.94 |
| Flu-like symptoms | -1.58 (1.25) | .210 | -4.06; 0.91 |
| Number of persons in household | -0.16 (0.23) | .494 | -0.62; 0.30 |
| Working in home office | 0.21 (0.68) | .756 | -1.15; 1.58 |
| Negative outcome expectancies at D0 | 0.15 (0.38) | .682 | -0.59; 0.90 |
| Within-level risk perception | -0.20 (0.21) | .348 | -0.61; 0.22 |
| Between-level risk perception | 0.42 (0.61) | .497 | -0.80; 1.64 |
| Situations as selected in the intervention (0 = not selected; 1 = selected) | | | |
| After coming home | -1.31 (0.77) | .094 | -2.86; 0.23 |
| After (longer) smartphone use | **1.70 (0.79)** | **.035** | 0.12; 3.28 |
| Before (preparing) a meal | 0.15 (0.86) | .866 | -1.56; 1.85 |
| Before snacking | 1.46 (0.99) | .145 | -0.51; 3.43 |
| Random effect | **Variance (*SE*)** | ***p*** |  |
| Intercept (at study start, D0) | 0.22 (10.80) | .984 |  |

*Note*. Analyses refer to *n* = 88 participants with 310 observations. *SE* = standard error. Significant predictions in bold. Regarding random effects, only a random intercept could be modelled. The inclusion of more random effects resulted in model non-convergence.
